# Population mobility data provides meaningful indicators of fast food intake and diet-related diseases in diverse populations

**DOI:** 10.1038/s41746-023-00949-x

**Published:** 2023-11-15

**Authors:** Abigail L. Horn, Brooke M. Bell, Bernardo García Bulle Bueno, Mohsen Bahrami, Burçin Bozkaya, Yan Cui, John P. Wilson, Alex Pentland, Esteban Moro, Kayla de la Haye

**Affiliations:** 1 Information Sciences Institute and Department of Industrial and Systems Engineering, Viterbi School of Engineering, University of Southern California, Los Angeles, CA USA; 2 Department of Population and Public Health Sciences, Keck School of Medicine, University of Southern California, Los Angeles, CA USA; 3 Department of Chronic Disease Epidemiology, Yale School of Public Health, Yale University, New Haven, CT USA; 4 Institute for Data, Systems, and Society, Massachusetts Institute of Technology, Cambridge, MA USA; 5 Sabanci Business School, Sabanci University, Istanbul, Turkey; 6 Los Angeles County Department of Public Health, Los Angeles, CA USA; 7 Spatial Sciences Institute, Dornsife College of Letters, Arts and Sciences, University of Southern California, Los Angeles, CA USA; 8 Departments of Civil & Environmental Engineering and Computer Science, Viterbi School of Engineering, University of Southern California, Los Angeles, CA USA; 9 Departamento de Matemáticas & GISC, Universidad Carlos III de Madrid, Leganés, Spain; 10 Institute for Food System Equity, Center for Economic and Social Research, University of Southern California, Los Angeles, CA USA

**Keywords:** Risk factors, Obesity, Geography, Computational science

## Abstract

The characteristics of food environments people are exposed to, such as the density of fast food (FF) outlets, can impact their diet and risk for diet-related chronic disease. Previous studies examining the relationship between food environments and nutritional health have produced mixed findings, potentially due to the predominant focus on static food environments around people’s homes. As smartphone ownership increases, large-scale data on human mobility (i.e., smartphone geolocations) represents a promising resource for studying dynamic food environments that people have access to and visit as they move throughout their day. This study investigates whether mobility data provides meaningful indicators of diet, measured as FF intake, and diet-related disease, evaluating its usefulness for food environment research. Using a mobility dataset consisting of 14.5 million visits to geolocated food outlets in Los Angeles County (LAC) across a representative sample of 243,644 anonymous and opted-in adult smartphone users in LAC, we construct measures of visits to FF outlets aggregated over users living in neighborhood. We find that the aggregated measures strongly and significantly correspond to self-reported FF intake, obesity, and diabetes in a diverse, representative sample of 8,036 LAC adults included in a population health survey carried out by the LAC Department of Public Health. Visits to FF outlets were a better predictor of individuals’ obesity and diabetes than their self-reported FF intake, controlling for other known risks. These findings suggest mobility data represents a valid tool to study people’s use of dynamic food environments and links to diet and health.

## Introduction

Food environments, the spaces where people acquire and consume food, impact diet and related diseases (i.e., nutritional health)^1^. To date, research has focused on predefined local and static food environments, largely of the home neighborhood^2,3^. Their features (e.g., the availability of fast food outlets) can predict nutritional health^1^ although findings are mixed^4–6^. A growing proportion of food acquisition occurs miles from our homes^7^, therefore the limited focus on static food environments may be one cause of these mixed results.

A major gap in the literature is evidence of the dynamic food environments people are exposed to in their daily routines (i.e., their “activity space”^8^), the food outlets they visit, and how these mobile food environments impact dietary intake and health. With the availability of big data on human mobility (i.e., geolocations captured by people’s smartphones), population-level research on the food outlets that people have access to and visit given their daily movements is now possible. Some studies (often *n* < 100) have begun to use GPS tracking technologies to continuously observe how people navigate their environment to acquire food over relatively brief time periods (i.e., 1 week)^9,10^. However, to our knowledge, large-scale mobility data has not been used to study the relationship between people’s dynamic, mobile food environments and their food behaviors and dietary outcomes over extended time intervals.

A critical first step is to investigate whether visits to food outlets observed in population-level mobility data provide meaningful indicators of dietary intake and diet-related disease. Establishing the link between visits to food outlets observed in mobility data and nutritional health is also an important methodological issue. Eating behaviors are notoriously hard to accurately measure, traditional self-report-based assessment methods have well-established biases^11,12^, and surveillance data are limited in terms of both assessment frequency and spatial areas covered^13,14^. It would therefore be valuable to identify a data source that is passively and continuously collected to complement traditional dietary surveillance measures.

This study addresses these gaps by investigating the relationship between food-seeking behaviors observed in mobility data and dietary intake and diet-related disease from health survey data. We focus on fast food (FF) specifically because FF intake is linked to disease risk^15^, makes up 16% of Americans’ caloric intake^7^, and because FF outlets are plentiful and densely clustered in ‘food swamps’ that are concentrated in communities with the greatest health disparities^16^. First, we utilize a large mobility dataset from Los Angeles County (LAC), U.S.A., to generate neighborhood-level measures of visits to FF outlets as proxies for visits to FF outlets by the residents of those neighborhoods. We link the neighborhood-level measures to individual respondents from a health survey of a representative sample of LAC adults and test whether the neighborhood-level measures are associated with respondents’ self-reported FF intake, obesity, and diabetes. The first objective is to determine whether neighborhood-level visits to FF outlets are meaningful and significant indicators of respondents’ self-reported FF intake. The second objective is to determine whether neighborhood-level visits to FF outlets are a significant predictor of respondents’ obesity and diabetes, and a comparable or better predictor than self-reported FF intake. We find that neighborhood-level measures of visits to food outlets observed in mobility data strongly and significantly correspond to respondents’ self-reported FF intake, obesity, and diabetes, and are furthermore a better predictor of obesity and diabetes than their self-reported FF intake, controlling for other known risks. These findings suggest that mobility data represents a valid tool to study people’s use of dynamic food environments and links to diet and health, with applications ranging from behavioral health monitoring to population-scale investigations into how food environments influence nutritional health.

## Results

### Health and demographic data study population

Individual-level measures of FF intake and diet-related disease come from the 2011 Los Angeles County Health Survey (LACHS), a population‐based dual frame (landline and cellular) telephone survey conducted by the Los Angeles County Department of Public Health (LACDPH)^17^. Our analytic sample included 5447 adults (18 or older) with residential information, without missing data on any study variable, and not living in areas designated as ‘rural’^18^. When comparing the full (*n* = 8036) and analytic samples of LACHS respondents (Table 1), we found small (1–3%) but statistically significant differences in age group, gender, race and ethnicity, and household income level. The sample also differed on self-reported FF intake frequency, a four-category variable coded as never, infrequent (<once per month), moderate (≥once per month to <once per week), and frequent (≥once per week). Of the analytic sample, 17.3% reported never eating FF, 19.1% reported infrequent intake, 26.9% reported moderate intake, and 36.7% reported frequent intake; 24.8% had obesity (having a Body Mass Index, BMI ≥ 30); and 11.1% had diabetes.

**Table 1:** Demographic, diet, and diet-related disease characteristics in the full and analytic samples of participants of the 2011 Los Angeles County Health Survey (LACHS).

	Participants, no. (%)		
Characteristic	Full sample (*n* = 8036)	Analytic sample (*n* = 5447)	*P* value
Age			0.005
18–24	596 (7.4%)	467 (8.6%)	
25–29	438 (5.5%)	341 (6.3%)	
30–39	1204 (15.0%)	878 (16.1%)	
40–49	1596 (19.9%)	1063 (19.5%)	
50–59	1674 (20.8%)	1118 (20.5%)	
60–64	748 (9.3%)	464 (8.5%)	
65 or over	1780 (22.2%)	1116 (20.5%)	
Gender			0.044
Female	4863 (60.5%)	3201 (58.8%)	
Male	3173 (39.5%)	2246 (41.2%)	
Race and ethnicity			0.002
White	3414 (43.4%)	2257 (41.4%)	
Hispanic/Latino	2769 (35.2%)	2050 (37.6%)	
Black/African American	784 (10.0%)	584 (10.7%)	
Asian	728 (9.3%)	432 (7.9%)	
Multiracial/other	174 (2.2%)	124 (2.3%)	
Unknown	167	0	
Education			0.5
Less than high school	1385 (17.4%)	942 (17.3%)	
High school	1370 (17.2%)	965 (17.7%)	
Some college or trade school	2007 (25.2%)	1409 (25.9%)	
College or post graduate degree	3206 (40.2%)	2131 (39.1%)	
Unknown	68	0	
Income			0.034
Low	2980 (37.1%)	2119 (38.9%)	
High	5056 (62.9%)	3328 (61.1%)	
Fast food intake frequency			0.014
Never	1552 (19.4%)	944 (17.3%)	
Infrequent	1540 (19.3%)	1040 (19.1%)	
Moderate	2113 (26.9%)	1463 (26.9%)	
Frequent	2794 (34.9%)	2000 (36.7%)	
Unknown	37	0	
Obesity			0.072
No	5751 (76.6%)	4097 (75.2%)	
Yes	1757 (23.4%)	1350 (24.8%)	
Unknown	528	0	
Diabetes			>0.9
No	7125 (88.8%)	4841 (88.9%)	
Yes	895 (11.2%)	606 (11.1%)	
Unknown	16	0	

*P* values indicate *χ*^2^ test for the statistical significance of differences based on non-missing categories.

### Mobility data study population and measures of food outlet visits

We utilize a large, privacy-preserving population-scale mobility dataset collected by Spectus^19^ representing the geolocations from October 2016 - March 2017 (6 months) of 243,644 smartphone users living within LAC, representing 3.1% of the adult population^20^. We excluded users if they had fewer than two stays, or stops at any geographic location for 5 min or longer, resulting in an analytic sample of 234,995 users. Supplementary Table 1 provides statistics on the distribution of individual stays in the analytic sample. We find a total of 63,299,255 stays at locations within LAC, with a median (interquartile range [IQR]) number of stays per user of 172 (IQR, 93, 320). These stays were collected across a total of 16,009,417 observation days (Supplementary Table 4), with a median of 57 (IQR, 34, 90) days of observation for each user. Of these stays, 14,498,850 were at food outlets (22.9% of stays). Further details on the mobility data source and how stays were measured can be found in “Methods”.

Visits to food and FF outlets were identified by linking geolocated stays to a points of interest (POI) database from the Foursquare API^21^ in 2017, which provides the names and geolocations of 239,509 POI in LAC. Further details on this linkage are included in “Methods” and Supplementary Note: Mobility Data Processing. Food outlets were defined as any location where food might be sold (including restaurants, food retailers, and other locations), identified using Foursquare’s existing categorization taxonomy with manual checks. FF outlets were defined as the subgroup of limited-service restaurants serving menus predominantly containing ultra-processed and/or low-nutrient, energy-dense foods strongly linked to risk of obesity and type 2 diabetes (i.e., rich in highly processed meat and refined carbohydrate, sodium, total fat, saturated and trans fatty acids, cholesterol, and poor in essential nutrients and dietary fibers)^15^. We identified these FF outlets through a bottom-up search using a list of chain brands validated in previous nutritional health research as linked to disease risk^22^ (e.g., McDonald’s, Taco Bell, Pizza Hut). In total, we identified 53,588 food outlets and 4151 FF outlets in the Foursquare database. In comparison, the LAC Restaurant and Market Inventory^23^, which comprises Environmental Health permitted restaurants and markets in LAC that are inspected by the LAC Department of Public Health, contains a total of 40,600 restaurants and markets. See Supplementary Note: Detecting Food and Fast Food Outlets for further details on how food and FF outlets are measured.

We defined measures of visits to FF outlets at the level of each individual user, and then spatially aggregated and averaged these measures across users living within 272 LAC *Neighborhoods*^24^ (LACN) (Supplementary Fig. 1); see “Methods” for details on how LACN is defined. Data on food outlet visits from the mobility data sample needed to be aggregated into measures at an area level because privacy protections set out in the IRB protocols did not allow reporting of individual mobility user behavior. Aggregation was therefore the only way to relate measures of visits to food outlets to individual respondents in the LACHS health survey sample. Specifically, the mobility variables were linked as contextual variables to the analytic sample of LACHS survey respondents (*n* = 5447) based on their residential LACN (*n* = 247).

The neighborhood-aggregated food outlet visit measures can be seen as a proxy for the “typical” food outlet visit behaviors of residents of a neighborhood, given the following two assumptions: First, that the sample of smartphone users from the mobility data residing in a neighborhood is representative of that neighborhood’s population; and second, that adults living within a neighborhood have similar FF outlet visit patterns. To address the first assumption, we have established that the mobility user samples are representative of the population size of each neighborhood unit and demonstrate low bias across income classes (Supplementary Note: Mobility Data Representativeness).

The second assumption may present challenges given residents’ unique mobility patterns. However, the neighborhood measures are likely to be conceptually relevant for the following reasons: (i) Neighborhood-level measures representing (average) neighborhood-level behavior are commonly used in public health research and practice, such as census statistics, survey measures related to behavioral and metabolic health^25^, and measures of social determinants of health^26^. (ii) Neighborhood indicators assume some degree of homophily among residents based on evidence that people with similar characteristics, including food preferences^27^, self-select into similar neighborhoods. Similar exposures to social and structural factors within and beyond the neighborhood influence the behavior and health of neighborhood residents^8,28^. (iii) Neighborhood-level indicators are often used to identify priority populations for public health interventions and policies, such as interventions to improve neighborhood food environments through instituting FF bans or building supermarkets^29^.

Gaps in the measurement of each smartphone user (e.g., when phones are out of service) mean that we cannot measure behavior continuously for all users at all time points. We address this by defining percentage-based variables in which observations of FF outlet visits are relativized to other observables: Temporal frequency of FF outlet visits (FF visits/time) was defined as the percentage of observed periods (out of three possible daily periods: before 11:00 am, 11:00 am–4:00 pm, after 4:00 pm) in which a user visits at least one FF outlet in any LACN, out of the total number of observed periods for that user. Relative frequency of FF to all food outlet visits (FF visits/food) was defined as the percentage of the total number of visits to FF outlets for a user out of the total number of visits to any food outlet for that user. We also defined a covariate representing average mobility behavior as the average number of trips (trajectories between stays) per user per day (trips/day). These mobility variables were linked as contextual variables to the analytic sample of LACHS survey respondents (*n* = 5447) based on their residential LACN (*n* = 247).

**Fig. 1:**
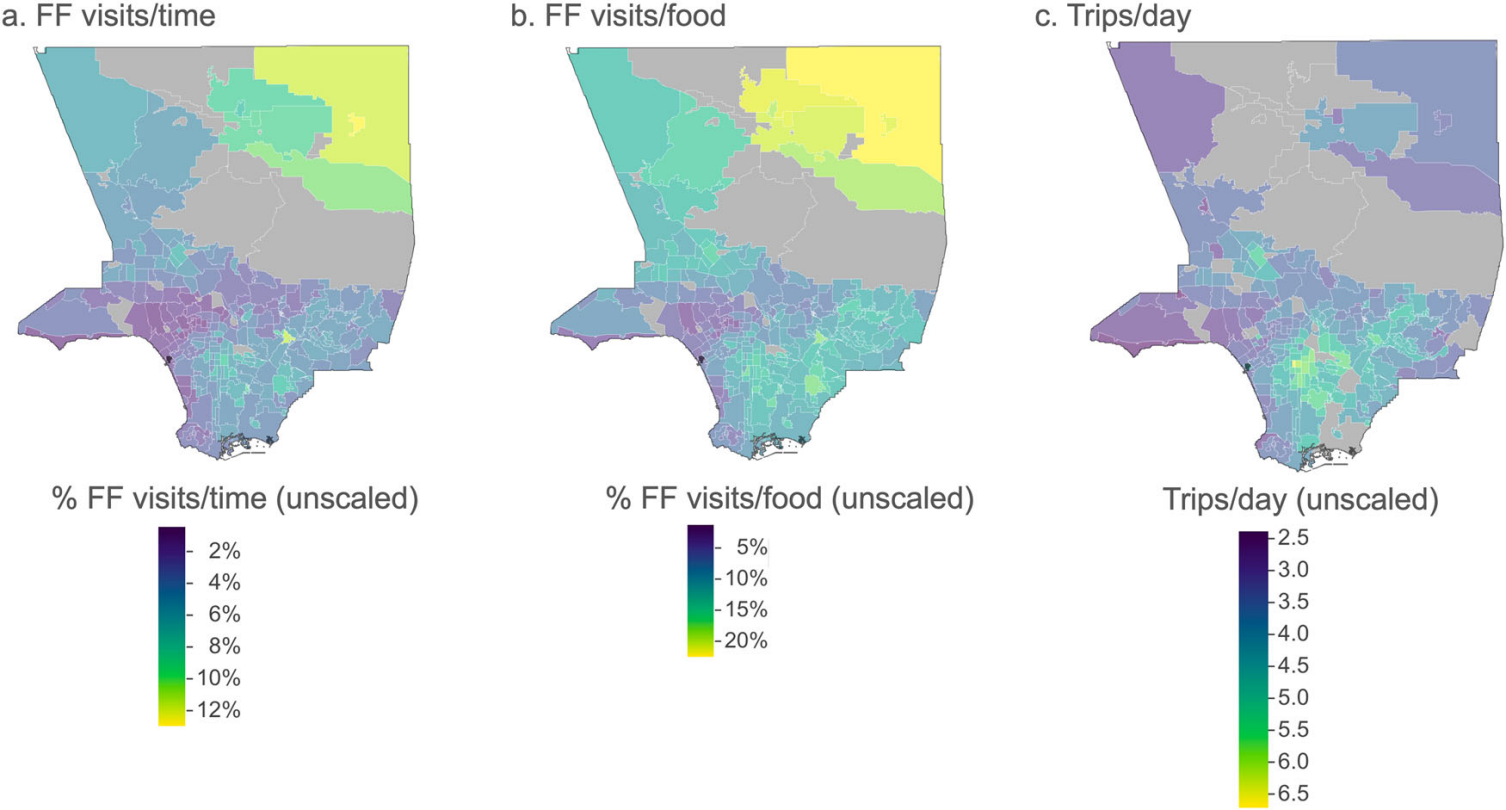
Geographic distribution of the unscaled mobility variables across Los Angeles County neighborhoods. *LACHS* Los Angeles County Health Survey, *FF* fast food. Figure represents the geographical distribution of the unscaled mobility variables across the 272 Los Angeles County Neighborhoods (LACN), before linkage to LACHS respondents. Areas with gray shading were predominantly rural neighborhoods. **a** FF visits/time represents the unscaled percentage of observed daily periods with visits to FF outlets; **b** FF visits/food represents the unscaled percentage of visits to all food outlets that were FF; and **c** Trips/day represents the unscaled number of trips per day; all averaged over all smartphone users with an estimated home residence within a LACN.

**Fig. 2:**
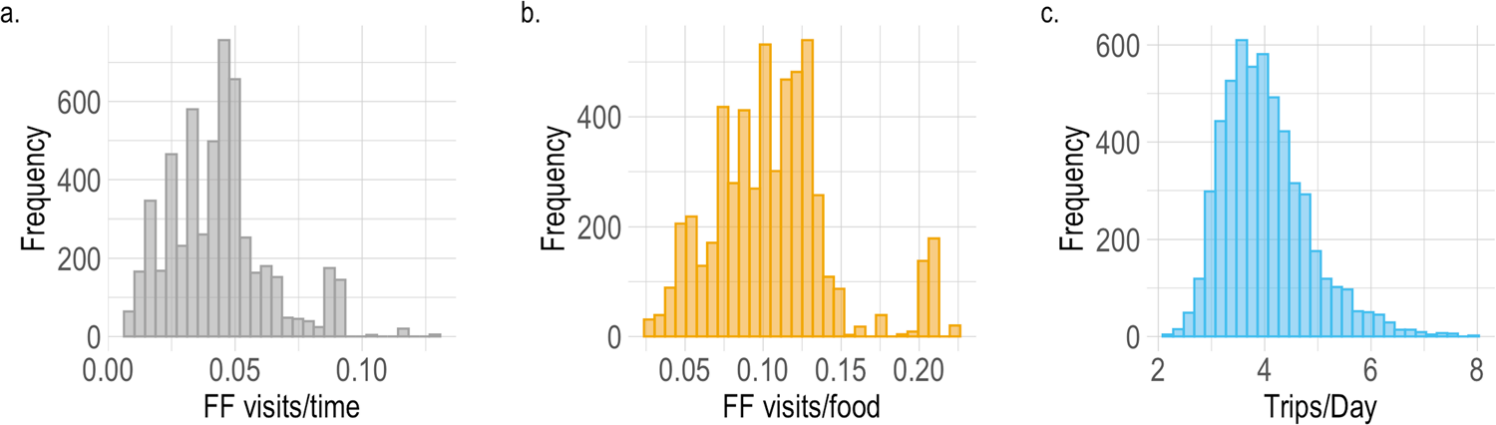
Histograms of the distribution of the unscaled mobility variables linked to LACHS respondents. *LACHS* Los Angeles County Health Survey, *FF* fast food. Histograms of the three mobility variables linked to LACHS respondents, unscaled. The range of variable values are presented in the *x* axis. Frequency counts in the *y* axis represent the number of LACHS respondents with a linked mobility variable at this binned interval. **a** FF visits/time represents the unscaled percentage of observed daily periods with visits to FF outlets; **b** FF visits/food represents the unscaled percentage of visits to all food outlets that were FF; and **c** Trips/day represents the unscaled number of trips per day; all averaged over all smartphone users with an estimated home residence within a LACN.

Across the mobility variables linked to LACHS respondents, the median (over respondents) percentage of observed daily periods in which smartphone users living in that LACN visited FF outlets (FF visits/time unscaled) was 4.3% (range, 1.0–13.0%); the median percentage of visits to any food outlets that were FF (FF visits/food unscaled) was 10.6% (range, 2.8–22.4%); and the median number of trips per day (trips/day unscaled) was 4.0 (range, 2.2–8.0). The variability of the neighborhood-level variables within any given LACN was much lower than across the LACN: the median (across LACN) variance of FF visits/time over smartphone users residing within a LACN was 0.7% (range of variance over LACN, 0.04–4.8%) and of FF visits/food was 2.0% (range of variance over LACN, 0.4–4.5%). Figure 1 shows geographic visualizations of the neighborhood-level variables before linkage to LACHS respondents, and Fig. 2 provides histograms of the distribution of the variables linked to LACHS respondents.

### Association between visits to FF outlets and FF intake frequency

In unadjusted multinomial logistic regression models predicting FF intake frequency, higher frequencies of FF outlet visits (aggregated across users in a LACN) were significantly associated with higher levels of individual FF intake for both FF mobility variables, investigated in separate models (Table 2). With a 10% increase in FF visits/time (i.e., a 1-unit increase in the scaled variable): relative to no FF intake, the odds of high FF intake increased by 35% (OR, 1.35; 95% CI, 1.28–1.42), of moderate FF intake increased by 26% (OR, 1.26; 95% CI, 1.19–1.33), and of low FF intake increased by 13% (OR, 1.13; 95% CI, 1.06–1.2). With a 10% increase in the frequency of FF visits/food: relative to no FF intake, the odds of high FF intake increased by 28% (OR, 1.28; 95% CI, 1.22–1.33), of moderate FF intake increased by 22% (OR, 1.22; 95% CI, 1.16–1.27), and of infrequent FF intake increased by 12% (OR, 1.12; 95% CI, 1.06–1.17). FF outlet visit variable effect estimates were not meaningfully changed after adding adjustment variables (AOR decreased by 1–4% from crude OR).

**Table 2:** Odds ratios for unadjusted and adjusted multinomial logistic regression analyses of the association between visits to fast food outlets observed in mobility data and self-reported fast food intake.

	Unadjusted analysis			Adjusted analysis		
Model	FF intake frequency, odds ratio (95% CI)			FF intake frequency, adjusted odds ratio (95% CI)		
	Infrequent	Moderate	Frequent	Infrequent	Moderate	Frequent
FF visits/time	1.13 (1.06–1.20)	1.26 (1.19–1.33)	1.35 (1.28–1.42)	1.12 (1.05–1.20)	1.22 (1.15–1.30)	1.30 (1.23–1.38)
FF visits/food	1.12 (1.06–1.17)	1.22 (1.16–1.27)	1.28 (1.22–1.33)	1.11 (1.06–1.17)	1.19 (1.14–1.24)	1.25 (1.19–1.31)

Reference group: no fast food intake. *P* < 0.001 for each estimated odds ratio. Each model estimated fast food intake frequency using the fast food visit variable listed in the Model column as the primary independent variable. Adjusted analyses consider age group, gender, race and ethnicity, educational level, and household income level. *CI* confidence interval, *FF* fast food.

### Association between visits to FF outlets and diet-related disease

In adjusted logistic regression models predicting diet-related disease, higher frequencies of FF outlet visits were associated with greater odds of obesity and diabetes. A 10% increase in FF visits/time was significantly associated with 16% (95% CI, 12–21%) greater odds of obesity and 15% (95% CI, 9–21%) greater odds of diabetes (Table 3). A 10% increase in FF visits/food was significantly associated with 13% (95% CI, 10–17%) greater odds of obesity and 11% (95% CI, 7–16%) greater odds of diabetes.

**Table 3:** Odds ratios for multivariable binary logistic regression analyses of the association between visits to fast food outlets and diet-related disease.

	Obesity		Diabetes	
Model	Adjusted odds ratio (95% CI)	*P* value	Adjusted odds ratio (95% CI)	*P* value
FF visits/time	1.16 (1.12–1.21)	<0.001	1.15 (1.09–1.21)	<0.001
FF visits/food	1.13 (1.10–1.17)	<0.001	1.11 (1.07–1.16)	<0.001
Self-report FF intake frequency (reference: never)				
Infrequent	1.06 (0.85–1.33)	0.621	1.08 (0.81–1.44)	0.594
Moderate	1.26 (1.03–1.55)	0.028	1.17 (0.89–1.54)	0.254
Frequent	1.63 (1.34–1.99)	<0.001	1.39 (1.08–1.81)	0.013

Each model includes the variable listed in the Model column as the primary independent variable. All models adjusted for age group, gender, race and ethnicity, educational level, and household income level. Adjusted odds ratios for the continuous variables (FF visits/time, FF visits/food) and the categorical variable (self-report FF intake frequency) are not directly comparable. *CI* confidence interval, *FF* fast food.

Obesity and diabetes were also predicted by self-reported FF intake. There was not a significant increase in the odds of obesity or diabetes between individuals with self-reported infrequent vs. no FF intake. However, individuals with moderate self-reported FF intake had a 26% (95% CI, 3–55%) greater odds of obesity than those with no FF intake. Individuals with frequent FF intake had a 63% (95% CI, 34–99%) greater odds of obesity and 39% (95% CI, 8–81%) greater odds of diabetes than those with no FF intake (Table 3). The odds ratios for the continuous variables and the categorical variable are not directly comparable.

These non-nested models were compared on the basis of Akaike weights, and transformations from raw Akaike Information Criterion (AIC) values to facilitate model comparison^30^. Comparing Akaike weights across the models of obesity, the probability that the model including FF visits/time was the best-fitting model was 0.10, including FF visits/food was 0.90, and including FF intake frequency was 3.6e-7 (Table 4). Comparing Akaike weights across the three models of diabetes, the probability that the model including FF visits/time was the best-fitting model was 0.69, including FF visits/food was 0.31, and including self-reported FF intake frequency was 2.3e-5. Thus, the mobility variables were the better predictor of obesity and diabetes, compared to self-reported FF intake frequency.

**Table 4:** Values of the Akaike Information Criterion (AIC) and Akaike weights calculated from binary logistic regression models of the association between visits to fast food outlets, self-report fast food intake frequency, and diet-related disease.

	Obesity		Diabetes	
Model	AIC	Akaike weight	AIC	Akaike weight
FF visits/time	5754.7	0.10	3353.8	0.69
FF visits/food	5750.2	0.90	3355.4	0.31
Self-report FF intake frequency	5777.9	3.6e-7	3374.4	2.3e-5

Each model includes the variable listed in the Model column as the primary independent variable. All models adjusted for age group, gender, race and ethnicity, educational level, and household income level. Each model’s Akaike weight can be interpreted as the probability that it is the best model out of the set of three candidate models. *AIC* Akaike information criterion, *FF* fast food.

### Sensitivity of findings to time gap between datasets

The LACHS and mobility datasets are from different years because FF intake was not assessed by the LACHS survey after 2011, while geolocation-based mobility data was not available before 2016, representing possible incompatibility between linked population samples. We evaluated the sensitivity of regression results to this time gap. Previous research on a U.S.-wide individual-level longitudinal sample has demonstrated stability of associations between neighborhood-level predictors and individual-level behavioral outcomes measured at different time points, if neighborhood characteristics most predictive of the outcomes are also stable over the intervening time period^31^. We tested whether this finding could apply to our own data by (i) evaluating the stability of neighborhood-level characteristics most predictive of FF intake (relating to ethnicity and income) across Census tract neighborhoods in the LAC study region between 2011–2017, using data from the American Community Survey^20^; and (ii) re-estimating regression models after removing the LACHS study participants residing in Census tracts with the greatest demographic change.

We found that Census tract-level ethnicity and income population characteristics (measured as the percentage of the population above 200% of the Federal Poverty Limit, FPL; that is Black or African American Alone; and that is Hispanic or Latino) remained relatively stable during the time gap between data collection for the LACHS (2011) and mobility data (2016–17). Supplementary Fig. 2 shows the distributions of the differences in these percentage-based characteristics between 2011 and 2017, which are approximately normally distributed and mean-centered at 0. Across all Census tracts, 95% demonstrated less than: a 16% change in the percentage of the population above 200% of the FPL; a 10% change in the population that is Black or African American Alone; and a 15% change of the population that is Hispanic or Latino.

We also found that regression effect estimates experienced little to no change after removing LACHS respondents in neighborhoods with the greatest demographic changes. We used two outlier detection methods to identify Census tracts demonstrating the largest change in any of the three neighborhood-level characteristics considered and removed LACHS respondents living in those neighborhoods from the analytic sample (details in “Methods”). In unadjusted multinomial regression models of FF intake frequency fit to the full analytic sample compared with models fit to the analytic sample subtracting out respondents living in outlier Census tracts, odds ratios changed by 2% or less for all categories for FF intake frequency and both methods of outlier identification and removal (Table 5). In adjusted logistic regression models of obesity and diabetes fit the full analytic sample compared with results from regression models fit the analytic sample subtracting out respondents living in outlier Census tracts, odds ratios for models of obesity changed by <1% and for diabetes by <2% (Table 6).

**Table 5:** Unadjusted Odds ratios from multinomial logistic regression models of fast food intake in sensitivity analyses to time change between 2011 and 2017.

	FF intake frequency, unadjusted odds ratio (95% CI)								
	Infrequent			Moderate			Frequent		
Model	Full sample	Method 1	Method 2	Full sample	Method 1	Method 2	Full sample	Method 1	Method 2
FF visits/time	1.13 (1.06, 1.20)	1.12 (1.04, 1.19)	1.11 (1.04, 1.19)	1.26 (1.19, 1.33)	1.28 (1.20, 1.36)	1.26 (1.19, 1.34)	1.35 (1.28, 1.42)	1.38 (1.30, 1.46)	1.35 (1.27, 1.43)
FF visits/food	1.12 (1.06, 1.17)	1.11 (1.05, 1.17)	1.11 (1.05, 1.17)	1.22 (1.16, 1.27)	1.24 (1.18, 1.30)	1.22 (1.16, 1.28)	1.28 (1.22, 1.33)	1.30 (1.24, 1.36)	1.28 (1.22, 1.34)

Reference group: no fast food intake. *P* < 0.001 for all estimated odds ratios. Each model estimated fast food intake frequency using the fast food visit variable listed in the Model column as the primary independent variable. Sensitivity tests fit regression models to the analytic sample subtracting out respondents living in outlier census tracts demonstrating the largest change in demographic variables between 2011 and 2017 according to the two methods. Method 1: Outliers are identified by the Tukey method as values more than 1.5 times the interquartile range from each of the quartiles for a variable, i.e., upper outliers are values of the distribution >Q3 + 1.5 × IQR and lower outliers are values <Q1–1.5 × IQR. Method 2: Outliers are identified as values of a variable above and below 2 standard deviations of the mean. For each method, we remove from the LACHS sample all respondents living in outlier census tracts. We then identified the union over outlier census tracts across the three measures, as some census tracts overlapped. Regression model results fit to the full analytic sample (full sample) are provided for comparison. *CI* confidence interval, *FF* fast food.

**Table 6:** Adjusted Odds ratios from binary logistic regression models of obesity and diabetes in sensitivity analyses to time change between 2011 and 2017.

	Full sample		Method 1		Method 2	
Model	AOR of obesity (95% CI)	AOR of diabetes (95% CI)	AOR of obesity (95% CI)	AOR of diabetes (95% CI)	AOR of obesity (95% CI)	AOR of diabetes (95% CI)
FF visits/time	1.16 (1.12, 1.21)	1.15 (1.08, 1.21)	1.17 (1.12, 1.22)	1.17 (1.09, 1.24)	1.17 (1.12, 1.22)	1.16 (1.09, 1.23)
FF visits/food	1.13 (1.10, 1.17)	1.11 (1.06, 1.16)	1.14 (1.09, 1.18)	1.12 (1.06, 1.17)	1.14 (1.10, 1.18)	1.12 (1.06, 1.17)

*P* < 0.001 for all estimated odds ratios. Each model includes the variable listed in the Model column as the primary independent variable. All models adjusted for age group, gender, race and ethnicity, educational level, and household income level. Sensitivity tests fit regression models to the analytic sample subtracting out respondents living in outlier census tracts demonstrating the largest change in demographic variables between 2011 and 2017 according to the two methods. Method 1: Outliers are identified by the Tukey method as values >1.5 times the interquartile range from each of the quartiles for a variable, i.e. upper outliers are values of the distribution >Q3 + 1.5 × IQR and lower outliers are values <Q1–1.5 × IQR. Method 2: Outliers are identified as values of a variable above and below 2 standard deviations of the mean. For each method, we remove from the LACHS sample all respondents living in outlier census tracts. We then identified the union over outlier census tracts across the three measures, as some census tracts overlapped. Regression model results fit to the full analytic sample (full sample) are provided for comparison. *AOR* adjusted odds ratio, *CI* confidence interval, *FF* fast food.

Sensitivity analyses were also conducted to examine whether the associations between the FF visit variables and obesity or diabetes were meaningfully impacted by users’ general mobility behavior (trips/day), a potential confounder associated both with these diseases and FF mobility behavior. Effect estimates were not impacted by controlling for general mobility behavior (Supplementary Table 2).

## Discussion

This study leverages large-scale population-level data on human mobility to study food environments and their connection with nutritional health. Using mobility data from LAC, we find strong and consistent evidence that visits to FF outlets aggregated over smartphone users residing in an LAC Neighborhood significantly correspond to the FF consumption of individuals living in those neighborhoods. Thus, passively observed visits to FF outlets via mobility data, aggregated at the neighborhood level, appear to be meaningful indicators of typical FF intake in a diverse, urban population.

Visits to FF outlets aggregated at the LACN level also predicted obesity and diabetes, two prevalent diet-related diseases, for individuals living in those neighborhoods. Moreover, model fit results show that mobility variables were comparable or better predictors of obesity and diabetes than individual self-reported FF intake, the latter a commonly used diet assessment tool in public health surveillance. Effect sizes held after controlling for individual-level sociodemographic factors and LACN-level general mobility behavior, suggesting these indicators uniquely represent visits to FF outlets rather than mobility alone.

The study finds agreement between the measures of *neighborhood-aggregated* FF outlet visit behavior, and self-reported FF intake and diet-related disease of individual health survey respondents residing in those neighborhoods. Conceptually, this suggests that the FF behaviors of the sample of smartphone users reflect those of the health survey sample and that both study samples are representative of the underlying residential population of the neighborhoods. These findings lend support to the two assumptions underlying the use of the neighborhood-aggregated measures of FF outlet visits as proxies for the “typical” FF outlet visit behaviors of residents of a neighborhood: (i) that population-scale mobility data can be used to construct representative samples of the behavior of the underlying population, and (ii) that adults living within a neighborhood have sufficiently similar food outlet visit patterns for neighborhood-aggregated measures to meaningfully represent the behaviors of individuals living in those neighborhoods. The second assumption is additionally supported by the finding that the variability in FF outlet visits across mobility users living within a neighborhood was much lower than the variability in aggregated FF outlet visits across neighborhoods.

In addition to the suitability of using neighborhood-aggregated measures of FF outlet visits as proxies for residents’ visits and ultimately for FF intake, several additional factors may explain the strength of our findings. First, measures of food behaviors observed directly from smartphone-captured mobility data may be less prone to measurement error than self-reported food intake, since mobility data captures behavior continuously, over months (or potentially longer), recording more detail of behavioral patterns than can be reliably assessed by self-reported recall^32,33^. Second, beyond the assumption that individuals with similar food preferences *self-select* into similar neighborhoods^27^, social feedback regarding eating behavioral norms may *influence* the eating behaviors of neighborhood residents. It is well-established that eating behavior is influenced by social and cultural factors and strengthened by propinquity^34^. This “social signal”^35^ may be captured by the aggregated measures of visits to FF outlets, providing additional prediction of disease risk. Third, the FF visit indicators may be picking up other neighborhood-level risk factors for these diseases, such as area-level deprivation.

This study has limitations. Its findings are prone to ecological fallacy, since we use aggregated area-level estimates of behaviors assumed to be typical of residents in those areas and investigate how these correspond to individual health behaviors and outcomes. Summarizing mobility features at the LACN (or other spatial) level will obscure individual or group differences, for example, differential visits to FF outlets based on gender or other demographics^36^. However, as noted, the validation of all study objectives suggests that this assumption may have been appropriate in the case of FF outlet visit measures.

Mobility variables were aggregated at the LACN level because (i) LACN boundaries were designed to represent similar groups of people^24^; (ii) this was the smallest spatial area over which we could demonstrate that our mobility user sample achieved broad geographic representation of the underlying population, and (iii) we could demonstrate that the variability of the mobility measures within was much smaller than across this chosen area. More highly-resolved aggregation should now be possible, since human mobility data available in 2022 is more highly sampled than in 2017, enabling aggregation of mobility variables over smaller areas while still protecting user privacy. Future analyses will be needed to explore the generalizability of this study’s findings when using mobility variables summarized over more refined levels of spatial aggregation. We also note that although the LACN is specific to LAC, the “neighborhood” unit is generalizable to other city contexts. For example, New York City has a similar mapping in the Neighborhood Tabulation Areas (*n* = 195) (https://www1.nyc.gov/site/planning/data-maps/nyc-population/geographic-reference.page); Chicago has a designation of 77 community areas for statistical and planning purposes, as well as 200 neighborhoods (https://www.chicago.gov/city/en/depts/dgs/supp_info/citywide_maps.html). For any given study, researchers will need to determine the best spatial level to aggregate mobility measures based on the data sampling and the research context. Future research will also be needed to explore the generalizability of our study findings to these different city contexts, which will likely differ in the spatial structure of food environments and patterns of food environment access.

An additional limitation is a potential bias in the smartphone user sample. Although smartphone users constituted 83% of the U.S. adult population in 2017^37^, they represent a subset of the population that has some uneven representation across sociodemographic groups (e.g., low-income, older, and non-white^38^). Quantifying these biases in mobility data is challenging since demographic information is not available to individual smartphone users to protect privacy. We have taken several steps to investigate and address the representativeness of the sample to the overall population: (i) we establish that our sample sizes are representative of the population size in each LACN, and (ii) in our previous work on this dataset, we demonstrated low bias across income classes by imputing this characteristic for each user based on their mobility-observed shopping behaviors^39^ (Supplementary Note: Mobility Data Representativeness). Different study designs will be necessary to investigate to what extent inferences derived from smartphone users are generalizable to all populations.

We have linked two different study populations and timeframes, which leads to potential sources of incompatibility. However, previous research on a U.S.-wide individual-level longitudinal sample has demonstrated that the power of neighborhood-level features to predict individual-level behavioral outcomes measured at a different time point is stable if neighborhood characteristics most predictive of the outcomes are also stable^31^. We evaluated the stability of neighborhood-level characteristics relating to ethnicity and income that are highly predictive of FF intake in our own data between 2011–17 and found very little change. Furthermore, regression effect estimates were not impacted when neighborhoods demonstrating the greatest change in these characteristics were removed. Together, these analyses suggest that FF outlet visit effect estimates were most likely not meaningfully affected by the time gap between dataset collection.

A limitation of the mobility data is the under-sampling of visits to FF outlets due to the gaps in measurement of each smartphone user (e.g., when phones are out of service). We address this by defining percentage-based variables in which observations of FF outlet visits are relativized to other observables (e.g., all food outlet visits), but there may be measurement error that cannot be quantified. Separately, the individual-level FF intake measures from the LACHS may be subject to biases common to self-report food frequency measures^11^.

We have taken several approaches to establish the robustness of findings to our methods to attribute geolocations to particular POI (Supplementary Note: Mobility Data Processing), and to define, detect, and appropriately label food and FF outlets (Supplementary Note: Detecting Food and Fast Food Outlets). However, there may be limitations to our ability to detect visits to certain food outlets, such as those in particularly dense urban areas or multi-story or multi-purpose buildings (e.g., malls) where FF outlets may be concentrated. Additionally, because we only detect visits greater than 5 min in duration, we may miss very brief FF outlet visits (e.g., drive-through).

A limitation of our study design is not knowing whether people consumed fast food when they visited an FF outlet, or the nutritional quality of the food they ordered. Our results suggest individuals were consuming FF during these visits because there is high correspondence with self-reported frequency of FF intake. Still, variability in nutritional content across menus and in food choices across patrons could serve to weaken the results of the association between FF outlet visits and diet-related disease. We partially addressed this limitation in our study design by focusing our definition of FF outlets on the subgroup of limited-service outlets serving menus predominantly containing ultra-processed and/or low-nutrient, energy-dense foods strongly linked to risk of obesity and type 2 diabetes. While the menus of this subgroup of outlets carry some “healthy” items (i.e., characterized by minimally processed ingredients, or using lower added sugars and saturated fats), extensive research and industry sales reports have documented that these “healthier options” are not what is typically consumed or purchased at FF outlets^40–44^. In ongoing work, we are developing methods to more realistically characterize the diversity of the nutritional quality of foods served at FF (and other types of) restaurants by analyzing digital menu data^45^. We also note that the variability in foods offered and ordered at FF outlets should not impact the relationships we observe between FF visits observed in the mobility data and self-reported FF intake, as the latter captures self-reported consumption of foods from FF restaurants, regardless of their type or nutritional quality (captured by the survey question “*How often do you eat any food, including meals and snacks, from a fast food restaurant like McDonald’s, Taco Bell, Kentucky Fried Chicken, or another similar type of place?*”

A further limitation is that our study focuses on in-person visits to FF outlets (including both sit-down and take-out meals) and does not account for fast food accessed via food delivery. However, this is unlikely to be a major limitation to the methods or translational value of the findings because food delivery represents a small fraction of overall food service sales, and a smaller fraction at the time our data were collected in 2016–17 than currently (2022); specifically an estimated 6.9% of overall food service sales in 2016, up to a projected 8.9% in 2022^46,47^. The mobility data we analyze in this study also illustrates the high prevalence of visits to physical food outlets across this large urban population, with 22.9% of observed visits to any place outside the home constituting visits to food outlets.

These limitations notwithstanding, our findings demonstrate compelling evidence that neighborhood-aggregated measures of visits to FF outlets passively collected from a diverse, urban sample of smartphone users provide strong and significant indicators of FF intake for people living in those neighborhoods. They also provide strong predictors of diet-related disease that are comparable to or better than self-reported FF intake, a standard measure in population nutrition research. Mobility data is objective and captured passively and continuously over long periods of time without recall bias, making it a convenient, information-rich means of gathering population-level food behaviors that are notoriously hard to measure. The results of our study suggest that this data source may represent a valid tool to complement population survey surveillance efforts for this and possibly other eating and health behaviors at small-area levels.

More broadly, this study introduces human mobility data as an untapped but useful resource for future investigations into how people of diverse backgrounds move around to dynamically use food environments, including and beyond the home neighborhood, and how this links to their diet and health. Studies involving mobility data might include: re-defining notions of “food deserts” and “food swamps” to account for lived environments beyond the home neighborhood; investigating routine behaviors that determine spatio-temporal accessibility to different types of food environments; using “natural experiments” (e.g., users who change home or food environments) to identify causal mechanisms linking features of food environments and eating behaviors; and developing more effective policies and interventions on food environments that take routine behaviors beyond the home neighborhood into consideration^48^.

## Methods

### Individual health and demographic data sources and measures

Individual-level measures of FF intake, diet-related disease, and sociodemographics, come from the 2011 Los Angeles County Health Survey (LACHS), a population‐based dual frame (landline and cellular) telephone survey conducted by Los Angeles County Department of Public Health (LACDPH). It collects data from representative samples of adults and children living within LAC, on topics such as health conditions and behaviors, sociodemographics, and home residence. Our study uses data from the Adult Survey module, which includes 8036 LAC residents who are 18 years and over. Detailed study protocols are available from LACHS^17^.

All variables analyzed in this study were self-reported to the LACHS survey-taker, and some were recorded from the measure coded and shared by LACHS for ease of interpretability (see Supplementary Note: Recoding LACHS measures). FF intake frequency was coded as a four-category variable: never, infrequent (<once per month), moderate (≥once per month to <once per week), and frequent (≥once per week). Obesity (BMI ≥ 30) and Diabetes (having a diagnosis), were coded as binary variables (yes/no). Sociodemographic factors included age group, gender, race and ethnicity, education level, and household income level. Respondents’ LACN was derived from their home address.

We excluded participants who: were missing residential information (*n* = 2007), lived in areas designated as ‘rural’^18^ (*n* = 104), or had missing data for any study variable (*n* = 478). The final analytic sample was 5447 participants.

### Individual geolocation (mobility) data source and aggregated measures

Geolocation (i.e., mobility) data were collected by Spectus^19^, a location-based services company that maintains anonymized geospatial datasets on human mobility by aggregating data across smartphone applications from mobile phone devices, and shared through their Social Impact Program. The dataset consists of anonymized records of GPS locations from individual adult (≥18 years) smartphone users who opted in to provide access to their GPS location data anonymously through a General Data Protection Regulation and California Consumer Privacy Act-compliant framework. Users across all major smartphone device operating systems (e.g., iOS, Android, Windows) are represented. The dataset includes 243,644 users estimated to live within LAC (explained below) between October 2016–March 2017 (6 months).

The data consists of geolocation “pings” identifying the location of a given smartphone. The majority of these pings are recorded every 5–15 min, although they can be more or much less frequent (e.g., if a phone is out of range). Each ping contains the GPS location of the phone (latitude and longitude), timestamp, an anonymous (encrypted and hashed) identifier unique for all smartphone users, and an estimate of its *horizontal positioning accuracy* (defined as the radius that provides a 68% (1*σ*) confidence that the device is within that radius)^49^. Median ping accuracy was 21 m (Supplementary Fig. 3). No other individual information was available to users.

When a user spends significant time in a single location, measurement uncertainty will cause a number of pings to be scattered around the actual location. To map these events to a single “stay” with an accurate time and location, we use the Infostop algorithm^50^, which clusters consecutive events together if the maximum distance from their centroid, computed as the median of the pings’ latitudes and longitudes, is less than some roaming distance, *d*^*roam*^. The first and last ping mark the start and end time of the stay. At least two subsequent events need to be observed within *d*^*roam*^ to be considered a stay. We use *d*^*roam*^ = 50 m, and set the minimum duration of a stay to 5 min. We excluded users if they had fewer than two stays at any location over the 6 months, resulting in an analytic sample of 234,995 users with over 63 million observed stays.

Visits to food and FF outlets were identified by linking stays to the Foursquare POI database^21^ obtained in 2017, which provides the names and geolocations of 239,509 POI in LAC. We attributed each stay to the closest POI in our dataset, calculated from the centroid of the user’s stay to the centroid of the spatial polygon of the POI, which is a validated approach for inferring visited POI from passively collected smartphone mobility data^51^. We choose only the closest POI within a radius of a thresholding maximum distance, *d*^*max*^, which we set at 200 m. Robustness tests demonstrated that calculated mobility variables and study findings were robust to the choice of *d*^*max*^. See Supplementary Note: Mobility Data Processing for further details on attributing stays to POI and robustness tests. Methods for defining how Food and FF outlets were measured are provided in Results, with further details found in Supplementary Note: Detecting Food and Fast Food Outlets.

Aggregation to an area level was necessary because privacy protections set out in the IRB protocols did not allow reporting of individual mobility user behavior, and because this was the only way to link to the LACHS health data. The LAC *Neighborhoods*^24^ (LACN) level was chosen because (i) we show that the variability in mobility variables calculated across users within each LACN was low (see “Results”); (ii) the LACN was the smallest administrative area we could demonstrate that our mobility user sample achieved broad geographic representation of the underlying population, evaluated through comparison with U.S. Census data (Supplementary Note: Neighborhood-level aggregation of mobility-derived variables); and (iii) because the area has social-cultural meaning: LACNs were designed via a community-sourced project, led by the Los Angeles Times, with the goal of mapping LAC communities “with more similar groups of people”^24^ (Supplementary Note: Background on LACN). The median population size in a LACN was 27,499 (IQR, 12,961–53,124), while the median number of smartphone users was 468 (IQR, 243–914).

We estimated the home LACN for each user as that in which the majority of their activity between 10:00 pm–6:00 am occurred. We calculated the mobility measures for FF visits/time, FF visits/food, and trips/day for each user over the 6-month observation period, as summarized in “Results” and described in formal detail in Supplementary Note: Constructing Mobility Variables. These three measures were averaged over all individual users with an estimated home residence within a LACN, and rescaled from 0–10 to enable comparison of effect sizes in regression analyses.

All study protocols were approved by the Institutional Review Boards (IRB) of the LACDPH, the University of Southern California, and the Massachusetts Institute of Technology. Where applicable, this study followed the Strengthening the Reporting of Observational Studies in Epidemiology (STROBE) reporting guidelines.

### Statistical analysis

**Fig. 3:**
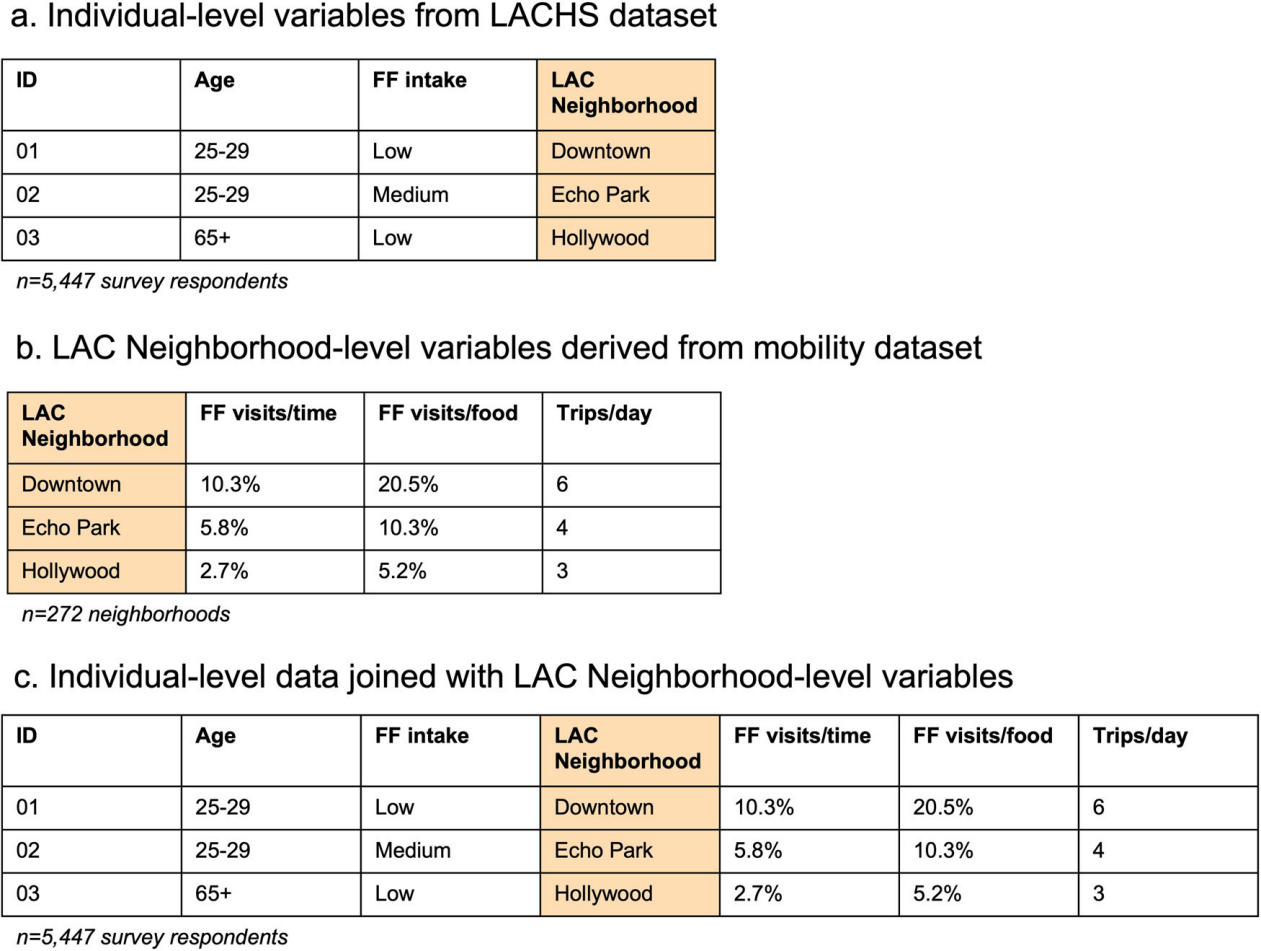
Visualization of process for linking the individual-level health survey data and the mobility-derived fast food outlet visit variables. *LACHS* Los Angeles County Health Survey, LAC Los Angeles County. **a** shows an example of three variables and a respondent ID from the individual-level LACHS dataset. **b** shows an example of three LAC Neighborhood-aggregated variables calculated from the individual-level Spectus mobility dataset and LAC Neighborhood ID. **c** shows an example of the LAC neighborhood-level variables linked as contextual variables to the individual-level LACHS dataset.

Mobility variables for LACN were linked as contextual variables to the analytic sample of LACHS survey respondents (*n* = 5447) based on their estimated residential LACN (*n* = 247 unique LACN across the analytic sample) (Fig. 3). Descriptive characteristics of the analytic sample with linked mobility variables were computed and compared to those in the full sample (*n* = 8036) using *χ*^2^ tests.

Logistic regression models at the individual LACHS respondent level with linked LACN level mobility variables (*n* = 5447) were generated to test the study objectives. For the first objective, unadjusted and adjusted multinomial logistic regression models were used to estimate the odds ratios (ORs) and adjusted ORs (AORs) for the association between the two FF outlet visit variables (IV), and self-reported FF intake frequency as the dependent variable (DV) in separate models for each IV. The multivariable models were adjusted for individual-level sociodemographic risk factors (see Supplementary Note: Equations for Regression Models).

For the second objective, multivariable logistic regression models tested whether (i) FF visits/time (IV), (ii) FF visits/food (IV), and (iii) self-reported FF intake (IV) were associated with obesity (DV), and in separate models, with diabetes (DV). All models were adjusted for sociodemographic factors. Odds ratios for the categorical variable (FF intake frequency) and the continuous variables (FF visits/time and FF visits/food) cannot be directly compared. The non-nested models were therefore compared on the basis of Akaike weights, and transformations from raw AIC values to facilitate the interpretation of AIC model comparisons^30^. A model’s Akaike weight is interpreted as its probability of being the best out of a set of candidate models.

A two-sided *P* < 0.05 was considered statistically significant. Meaningful presence of confounding was evaluated as changes in estimated effect sizes of >10%. Mobility data were analyzed in Python; LACHS data and statistical analyses were conducted using R software, version 3.6.3.

### Sensitivity analysis

Relating measures of mobility to food outlets in 2016/17 with responses from a 2011 health survey represents a possible source of error in our approach. We investigated the sensitivity of study findings to this time gap. Previous research by Chetty et al. (2020)^31^ on a U.S.-wide individual-level longitudinal sample has demonstrated that the power of neighborhood-level features measured at the one-time point to predict behavioral outcomes for individuals living in those neighborhoods at different time points is stable (relative to models of predictors and outcomes measured at the same time) if neighborhood population characteristics most predictive of the outcomes are stable over the intervening time period^31^. Generalizing these findings to our study: if we find that (i) population characteristics were relatively stable over the intervening time period and (ii) regression effect estimates were not meaningfully different if models were refit after only neighborhoods with stable population characteristics were included, this would suggest that our regression results are similar to those produced from models using data measured at the same time period. We used this approach to evaluate the stability of our findings over the time period between the health survey and mobility data collection by (A) evaluating the stability of neighborhood-level characteristics highly predictive of FF intake (relating to ethnicity and income) in Census tracts within the LAC study region between 2011–17, and (B) re-estimating regression models after removing the LACHS study participants from the Census tracts demonstrating the greatest change.

The approach for part (A) used data from the 5-Year American Community Survey^20^ to evaluate the stability of three variables representing ethnicity and income, which are strong predictors of FF intake frequency: the percentage of the population in the Census tract living above 200% of the FPL, that is Hispanic or Latino, and that is Black or African American alone. The percentage of the population living above 200% of the FPL was used as a measure of income because it accounts for inflation and corresponds to the income measure available for LACHS study respondents.

We measured the distribution of differences in each of these three percentage-based measures between 2011 and 2017. We then identified LAC Census tracts that demonstrated the greatest amount of change (individually for each demographic variable) by two methods for outlier identification: (1) the Tukey approach, which defines outliers as values more than 1.5 times the interquartile range from each of the quartiles (Method 1); and (2) the distribution above and below two standard deviations of the mean (Method 2). Stability was analyzed by evaluating the absolute percentage change demonstrated by 95% of the Census tracts, i.e., identifying Z in *P*(|*X*_i_| < *z*) = 0.95, where *X*_i_ represents each of the three demographic characteristics analyzed.

The approach for part (B) involved re-estimating regression models after removing the LACHS study participants from the Census tracts demonstrating the greatest change and comparing to results on the full analytic sample. For each outlier identification method, we first removed the union of outlier Census tracts across the three measures and the LACHS respondents linked to these Census tracts. For Method 1 (Tukey method), 11.0% of Census tracts were removed, accounting for 12.4% of LACHS respondents; for Method 2 (2 standard deviations from the mean), 6.7% of Census tracts were removed as outliers accounting for 12.0% of LACHS respondents (Supplementary Table 3).

We then re-estimated the regression models presented in the main text after removing the linked LACHS study participants from these Census tracts. Regression analyses were rerun for six models representing the primary results analyzed in this study after removing LACHS respondents in the outlier Census tracts. These models examined the association between each of the two FF outlet visit measures observed from the mobility data, FF visits/time, and FF visits/food, and each of the three outcome measures analyzed, FF intake frequency, obesity, and diabetes. We adjusted for risk factors in the obesity and diabetes outcome models. We evaluated the percentage change in the regression odds ratios produced by fitting to the full analytic sample and the sample after subtracting out outlier census tracts according to the two methods. Percentage changes above 10% were considered meaningfully different.

Separately, we included sensitivity analyses to examine whether the observed relationships between visits to FF outlets and FF intake and diet-related disease are uniquely due to FF outlet visits, and not general mobility behavior irrespective of FF visits. Analyses were included to evaluate models that control for an indicator of general mobility behavior, trips/day.

### Reporting summary

Further information on research design is available in the Nature Research Reporting Summary linked to this article.

### Supplementary information

Supplementary Material

Reporting Summary

## Data Availability

Mobility data were shared by Spectus through their Social Impact. Conditions, limitations, and information on how to request data access can be found at https://spectus.ai/social-impact. The LACHS health survey data are available to investigators upon request and pending eligibility to access data governed by the Los Angeles County Department of Public Health Institutional Review Board. Other data used come from the 2017 5-Year American Community Survey, available at https://www.census.gov/programs-surveys/acs.

## Code availability

The underlying code for this manuscript is not publicly available due to the protection of the study data. Code may be made available upon reasonable request from the corresponding author, contingent upon eligibility to access study data as determined by the Los Angeles County Department of Public Health Institutional Review Board and mobility data from Spectus as noted in the Data Availability section.

## References

[1] Story M, Kaphingst KM, Robinson-O’Brien R, Glanz K (2008). Creating healthy food and eating environments: policy and environmental approaches. Annu. Rev. Public Health.

[2] Feng J, Glass T, Curriero F, Stewart W, Schwartz B (2010). The built environment and obesity: a systematic review of the epidemiologic evidence. Health Place.

[3] Leal C, Chaix B (2011). The influence of geographic life environments on cardiometabolic risk factors: a systematic review, a methodological assessment and a research agenda. Obes. Rev..

[4] Cobb L (2015). The relationship of the local food environment with obesity: a systematic review of methods, study quality, and results. Obesity.

[5] Dubowitz T (2015). Healthy food access for urban food desert residents: examination of the food environment, food purchasing practices, diet and BMI. Public Health Nutr..

[6] Chen, X. & Kwan, M. P. Contextual uncertainties, human mobility, and perceived food environment: the uncertain geographic context problem in food access research. *Am. J. Public Health* **105**, 1734–1737 (2015). 10.2105/AJPH.2015.302792 PMC4539815 26180982

[7] Saksena, M. J. et al. America’s eating habits: food away from home. EIB-196 (2018).

[8] James P (2016). Spatial energetics: integrating data from GPS, accelerometry, and GIS to address obesity and inactivity. Am. J. Prev. Med..

[9] Chaix B (2018). Mobile sensing in environmental health and neighborhood research. Annu. Rev. Public Health.

[10] Scully JY, Moudon AV, Hurvitz PM, Aggarwal A, Drewnowski A (2019). A time-based objective measure of exposure to the food environment. Int. J. Environ. Res. Public Health.

[11] Spruijt-Metz D (2018). Advances and controversies in diet and physical activity measurement in youth. Am. J. Prev. Med..

[12] McClung HL (2018). Dietary intake and physical activity assessment: current tools, techniques, and technologies for use in adult populations. Am. J. Prev. Med..

[13] Wang YC, DeSalvo K (2018). Timely, granular, and actionable: informatics in the public health 3.0 era. Am. J. Public Health.

[14] Shakeri Hossein Abad, Z. et al. Digital public health surveillance: a systematic scoping review. *npj Digit. Med.* **4**10.1038/s41746-021-00407-6 (2021). 10.1038/s41746-021-00407-6 PMC7930261 33658681

[15] Bahadoran Z, Mirmiran P, Azizi F (2015). Fast food pattern and cardiometabolic disorders: a review of current studies. Health Promot. Perspect..

[16] Cooksey-Stowers K (2017). Food swamps predict obesity rates better than food deserts in the United States. Int. J. Environ. Res. Public Health.

[17] Los Angeles County Department of Public Health. Los Angeles County Health Survey. http://www.publichealth.lacounty.gov/ha/hasurveyintro.htm.

[18] United States Department of Agriculture Economic Research Service. Rural-urban commuting area codes (Updated 7/3/2019). https://www.ers.usda.gov/data-products/rural-urban-commuting-area-codes.aspx (2014).

[19] Spectus Social Impact. https://spectus.ai/social-impact.

[20] United States Census Bureau. 2017 American community survey 5-year data. https://www.census.gov/programs-surveys/acs.

[21] Foursquare API. https://developer.foursquare.com/.

[22] Datar A, Mahler A, Nicosia N (2020). Association of exposure to communities with high obesity with body type norms and obesity risk among teenagers. JAMA Netw. open.

[23] Los Angeles County Department of Public Health. County of Los Angeles restaurant and market inventory. https://data.lacounty.gov/Health/COUNTY-OF-LOS-ANGELES-RESTAURANT-AND-MARKET-INVENT/jf4j-8it9.

[24] The Los Angeles Times Datadesk. Mapping L.A. Neighborhoods. http://maps.latimes.com/neighborhoods/.

[25] CDC. Behavioral Risk Factor Surveillance System. https://www.cdc.gov/brfss/index.html.

[26] PhenX Social Determinants of Health (SDOH) Assessments Collection. https://www.nimhd.nih.gov/programs/collab/phenx/.

[27] Boone-Heinonen J, Gordon-Larsen P, Guilkey DK, Jacobs DR, Popkin BM (2011). Environment and physical activity dynamics: the role of residential self-selection. Psychol. Sport Exerc..

[28] Sallis, J. F., Owen, N. & Fisher, E. B. Ecological models of health behavior theory, research, and practice. *In*: Health behavior and health education: theory, research, and practice, (2008).

[29] Mah, C. L., Luongo, G., Hasdell, R., Taylor, N. G. A. & Lo, B. K. A systematic review of the effect of retail food environment interventions on diet and health with a focus on the enabling role of public policies. *Curr. Nutr. Rep.* **8**, 411–428 (2019). 10.1007/s13668-019-00295-z PMC6904419 31797233

[30] Wagenmakers, E. J. & Farrell, S. AIC model selection using Akaike weights. *Psychon. Bull. Rev.* **11**, 192–196 (2004). 10.3758/bf03206482 15117008

[31] Chetty, R. et al. The opportunity atlas: mapping the childhood roots of social mobility. National Bureau of Economic Research Working Paper https://www.nber.org/papers/w25147 (2020).

[32] Riley, W. T. et al. Health behavior models in the age of mobile interventions: are our theories up to the task? *Transl. Behav. Med.* **1**, 53–71 (2011). 10.1007/s13142-011-0021-7 PMC3142960 21796270

[33] Spruijt-Metz D (2015). Building new computational models to support health behavior change and maintenance: new opportunities in behavioral research. Transl. Behav. Med..

[34] Herman, C. P., Polivy, J., Pliner, P. & Vartanian, L. R. *In*: Social influences on eating. 1st edn (2019).

[35] Galesic, M. et al. Human social sensing is an untapped resource for computational social science. *Nature* **595**, 214–222 (2021). 10.1038/s41586-021-03649-2 34194037

[36] Smith, L. G. et al. Comparing household and individual measures of access through a food environment lens: what household food opportunities are missed when measuring access to food retail at the individual level? *Ann. Am. Assoc. Geogr.* **112** (2021).

[37] Pew Research Center. Share of adults in the United States who owned a smartphone from 2011 to 2017, by location. https://www.statista.com/statistics/195003/percentage-of-us-smartphone-owners-by-geographic-location/.

[38] Coston, A. et al. Leveraging administrative data for bias audits: assessing disparate coverage with mobility data for COVID-19 policy. *In:* Proceedings of the 2021 ACM Conference on Fairness, Accountability, and Transparency. pp. 173–184, https://arxiv.org/abs/2011.07194 (2021).

[39] Moro E, Calacci D, Dong X, Pentland A (2021). Mobility patterns are associated with experienced income segregation in large US cities. Nat. Commun..

[40] Alot Living Team. The most popular fast food menu items. *Alot* https://living.alot.com/entertainment/the-most-popular-fast-food-menu-items–17399 (2020).

[41] Smith KJ (2009). Takeaway food consumption and its associations with diet quality and abdominal obesity: a cross-sectional study of young adults. Int. J. Behav. Nutr. Phys. Act..

[42] Bowman SA, Gortmaker SL, Ebbeling CB, Pereira MA, Ludwig DS (2004). Effects of fast-food consumption on energy intake and diet quality among children in a national household survey. Pediatrics.

[43] Paeratakul S, Ferdinand DP, Champagne CM, Ryan DH, Bray GA (2003). Fast-food consumption among US adults and children: dietary and nutrient intake profile. J. Am. Diet. Assoc..

[44] Prentice, A. M. & Jebb, S. A. Fast foods, energy density and obesity: a possible mechanistic link. *Obesity Rev.* **4**, 187–94 (2003). 10.1046/j.1467-789x.2003.00117.x 14649369

[45] Liu, I., Abeliuk, A., de la Haye, K. & Horn, A. L. A continuous indicator of food environment nutritional quality. https://www.medrxiv.org/content/10.1101/2021.11.24.21266841v1 (2021).

[46] Ahuja, K., Vishwa, Chandra., Victoria, Lord. & Curtis, Peens. Ordering in: the rapid evolution of food delivery. *McKinsey & Company* **148**, (2021).

[47] Solanki, A. & Saunders, N. Future of food: how ghost kitchens are changing the food landscape (Colliers International). (2019).

[48] Garcia Bulle Bueno, B. et al. You are where you eat: effect of mobile food environments on fast food visits. https://www.medrxiv.org/content/10.1101/2022.09.20.22280128v1 (2022). 10.1038/s41467-024-46425-2 PMC10937966 38480685

[49] Android Developer Reference: Location. https://developer.android.com/reference/android/location/Location.

[50] Hariharan, R. & Toyama, K. Project lachesis: parsing and modeling location histories. *Lecture Notes in Computer Science*. **3234**, 106–124 (2004).

[51] Cuttone, A., Larsen, J. E. & Lehmann, S. Inferring human mobility from sparse low accuracy mobile sensing data. UbiComp 2014 Adjunct Proceedings of the 2014 ACM International Joint Conference 731 on Pervasive and Ubiquitous Computing, September 13–17 (Seattle, WA, USA, 2014).

