# Supplementary material for: Population mobility data provides meaningful indicators of fast food intake and diet-related diseases in diverse populations
Source: NPJ Digit Med. 2023 Nov 15;6:208. doi: 10.1038/s41746-023-00949-x (PMC10651929; doi:10.1038/s41746-023-00949-x)
Supplement: Supplementary file 1 — Supplementary Material [file 41746_2023_949_MOESM1_ESM.pdf]

Supplementary Material:
Population mobility data provides meaningful indicators of fast food intake and diet related diseases in diverse populations

Abigail L. Horn; Brooke M. Bell; Bernardo Garcia Bulle Bueno; Mohsen Bahrami; Burcin Bozkaya; Yan Cui; John P. Wilson; Alex Pentland; Esteban Moro; Kayla de la Haye

Supplementary Notes

Supplementary Note 1. Recoding Los Angeles County Health Survey (LACHS) Measures
Supplementary Note 2. Mobility Data Processing
Supplementary Note 3. Identifying Food and Fasts Food Outlets
Supplementary Note 4. Los Angeles County Neighborhoods (LACN)
Supplementary Note 5. Neighborhood-Level Aggregation of Mobility-Derived Variables
Supplementary Note 6. Mobility Data Representativeness
Supplementary Note 7. Constructing Mobility Variables
Supplementary Note 8. Equations for Regression Models

Supplementary Tables

Supplementary Table 1. Statistics of the Distribution of Individual Stays in the Mobility Data Across the Analytic Sample of Smartphone Users
Supplementary Table 2. Logistic Regression Analyses of the Association Between Visits to Fast Food Outlets With Diet-Related Disease Adjusted for General Mobility Behavior
Supplementary Table 3. Number of Census Tracts and LACHS Respondents Removed After Identifying Outliers in Census Tract Demographic Change Between 2011 and 2017
Supplementary Table 4. Statistics of Stays at Each Value of Thresholding Maximum Distance
Supplementary Table 5. Unadjusted Odds Ratios of Self-Reported Fast Food Intake in Robustness Analyses of the Attribution of Stays Detected in Mobility Data to Points of Interest
Supplementary Table 6. Non-Food Primary Category and Subcategory Combinations Coded as Food Outlets in the Foursquare Dataset
Supplementary Table 7. List of Fast Food Outlet Names

Supplementary Figures

Supplementary Figure 1. Map of the Spatial Boundaries of the 272 Los Angeles County Neighborhoods
Supplementary Figure 2. Histograms of Differences in Three Census Tract Variables Between 2011 and 2017
Supplementary Figure 3. Frequency Distribution of the Accuracy of All Pings Generated by a Random Sample of 2,000 (0.8%) Smartphone Users in the Mobility Dataset
Supplementary Figure 4. Distribution of the Number of Smartphone Users in Analytic Sample Within Each Area for the Census Tract and Neighborhood Spatial Levels
Supplementary Figure 5. Correlation Between the Size of the Smartphone User Population Detected in Mobility Data and the Size of the Census Population at the Census Tract vs. Los Angeles County Neighborhood Levels
Supplementary Figure 6. Correlation Between Post-Stratified (Weighted) and Unweighted Fast Food Outlet Visit Variables

Supplementary Notes

1. Recoding Los Angeles County Health Survey (LACHS) Measures

The primary LACHS outcome variables used in this study were fast food (FF) intake frequency, obesity, and diabetes, and sociodemographic variables, and they were transformed and/or re-coded from the original measures for ease of interpretability. In addition to the text below, all measures coded by LACHS included categories for "do not know" or "refused". These responses were categorized as "unknown" and were not included in the analysis.

FF intake frequency: Respondents were asked, "How often do you eat any food, including meals and snacks, from a fast-food restaurant like McDonald's, Taco Bell, Kentucky Fried Chicken, or another similar type of place?" (1=4 or more times a week, 2=1-3 times/week, 3=less than once a week but more than once a month, 4=less than once a month, 5=never). Because of a small number of respondents in the "4 or more times a week" category (N=300, 3.7%), we re-coded this variable into the 4-category "FF intake frequency" variable analyzed in this study: never, infrequent (< once per month), moderate (≥ once per month to < once per week), and frequent (≥ once per week).

Obesity: Respondents reported their current height (inches) and weight (pounds), which was used to calculate Body Mass Index (BMI = kg/m^2). A variable for BMI status was coded by the LACHS across 4 categories based on the definitions from the National Heart, Lung, and Blood Institute (NHLBI)[1]: Obese (BMI ≥ 30), Overweight (25 ≤ BMI < 30), Normal Weight (18.5 ≤ BMI < 25), Underweight (BMI < 18.5). We re-coded this into the binary variable for "obesity" used in this study: 1=yes, 2=no.

Diabetes: Respondents were asked "Have you ever been told by a doctor or other health professional that you have diabetes or sugar diabetes [IF FEMALE, ADD: other than during pregnancy]?", coded by LACHS and used as the "diabetes" variable in this study: 1=yes, 2=no.

Sociodemographics:

(i) **Gender**: Gender was recorded by LACHS as male or female, used as the "gender" variable in this study.

(ii) **Age group**: "What is your age?" (numerical response in years) was recorded by LACHS with the categories: 18-24; 25-29; 30-39; 40-49; 50-59; 60-64; and 65 or over, used as the "age group" variable in this study.

(iii) **Race and ethnicity**: Race was measured by asking participants, "What is your race?" (white; Black/African American; Asian; Pacific Islander; American Indian/Alaskan Native; Hispanic/Latino; Other; Do not know; Refused). Hispanic origin was measured by asking participants, "Are you of Latino or Hispanic origin?" (yes, no). These two variables were coded by LACHS into a combination variable with 7 categories: Hispanic/Latino; white; Asian; Black/African American; Native Hawaiian or Pacific Islander; American Indian/Alaskan Native; Other, based on the following rules:

- If Hispanic/Latino mentioned at all, Hispanic/Latino was assigned.
- Else if Black/African American mentioned at all, African American was assigned.
- Else if Pacific Islander mentioned at all, Native Hawaiian or Other Pacific Islander (NHOPI) was assigned.
- Else if Asian mentioned at all, Asian was assigned.

- Else if white mentioned only, white was assigned.
- Else if American Indian/Alaska Native mentioned only, American Indian/Alaska Native (AI/AN) was assigned
- The remaining was assigned as Other.

We re-coded the combination variable into the 5-category "race and ethnicity" variable used in this study: Hispanic/Latino; white; Black/African American; Asian; and Multiracial/Other, which includes Pacific Islander, American Indian/Alaskan Native, do not know, and refused.

(iv) **Household income level**: Participants were asked for their annual household income. Responses were recoded by LACHS into a 4-category variable for household income level relative to the Federal Poverty Level (FPL): 0-99% FPL; 100-199% FPL; 200%-299% FPL; 300% or above FPL. We recoded this 4-category variable into the binary variable for "household income level" used in this study: low-income = <200% of the FPL, high-income = ≥200% FPL.

(v) **Education level**: "What is the highest level of school you have completed or the highest degree you have received?" was coded by LACHS into the 4-category variable: 1='Less than high school'; 2='High school'; 3='Some college or trade school'; 4='College or post graduate degree'.

LAC Neighborhood**:** Respondents reported their home address or nearest cross-streets to their home. Addresses were geocoded with street information to Census tracts, and then to LAC Neighborhood. When this information was not provided, the LAC Neighborhood was coded as missing.

2. Mobility Data Processing

2.1 Data collection

Geolocation (i.e., mobility) data were collected by Spectus[14], a location-based services (LBS) company that maintains anonymized geospatial datasets on human mobility by aggregating data across smartphone applications from mobile phone devices. Prior studies have used this particular individual-level data set to study inequality in use of urban spaces[2] and to model the impact of non-pharmaceutical interventions during the COVID-19 epidemic[3].

The data are collected from anonymized users who have opted-in to provide specific applications (apps) using LBS access to their GPS location. Permitting users have selected phone settings to allow LBS to be activated when specific apps are in use. Spectus collaborates with multiple smartphone app developers, supplying a patented software development kit (SDK) that provides a privacy-compliant path for anonymous opted-in users using these apps to share location data anonymously through a General Data Protection Regulation (GDPR) and California Consumer Privacy Act (CCPA) compliant framework. Devices across all major operational systems (e.g., iOS, Android, Windows) are included, although Android devices make up the majority of the data collected.

Data was shared in 2017 under a strict contract with Spectus through their Social Impact Program[4], which provides access to anonymized and privacy-protected mobility data for academic research and humanitarian initiatives only. All researchers were contractually obligated never to attempt to de-identify data, single out identifiable individuals, or link these data to third-party data about an individual. All study protocols were approved by the Institutional Review Boards (IRBs) of the Los Angeles County (LAC) Department of Public Health, the University of Southern California, and the Massachusetts Institute of Technology.

2.2 Data accuracy

Each device frequently broadcasts its location to a central server by sending its latitude, longitude, device ID, and the exact date and time of the event, which together represents a time-location *ping*. The ping data from Spectus also come with an estimate of the *horizontal positioning accuracy*, or accuracy, of each ping. For data collected from Android devices, accuracy is defined as the radius of 68% (1σ) confidence for the location, measured in meters (m). This means that if a circle is drawn centered at the latitude and longitude specified by the ping, with radius equal to the accuracy, there is a 68% probability that the true location is inside the circle[5]. The definition of horizontal positioning accuracy is similar for data collected from iOS (Apple) devices[6].

Supplementary Figure 3 presents a frequency distribution of the accuracy of all pings generated by a random sample of 2,000 (0.8%) of smartphone users in our dataset with accuracy levels ranging from 0 to 200m. The median accuracy of this distribution is 21m. An accuracy of 0 means that the accuracy level of the ping is < 1m. Pings with accuracy > 200m were not included in analysis for this study.

More generally, several studies have evaluated the accuracy of GPS data collection via crowdsourced Android and Apple devices. Accuracy has been found to be affected by various conditions including whether the smartphone is using GPS only or GPS combined with WiFi network, and the level of activity on that network; is within specific indoor locations; or outdoor locations (e.g., under leafy coverage, or in the presence of multiple multi-story buildings)[7,8]. A 2019 study found the average accuracy of location measurements in urban environments across two times of day and two times of year using an iPhone 6 to be between 7 - 13m; this is consistent with the accuracy range observed of recreation-grade GPS receivers (e.g., Garmin)[7]. While iPhone 6 was discontinued in 2016, it was still a prevalent model in use during the 2016-2017 data collection period. More recent iPhone models may have more highly resolved accuracy.

2.3 Detecting stays

When a user spends significant time at a single location, measurement uncertainty will cause a number of pings to be scattered around the actual location. To map these events to a single stay with an accurate time and location, we use the Infostop algorithm[9]. Extracting stays compresses ping trajectories into a series of stay points coded with the attributes latitude, longitude, start time, and end time. To extract the locations of stays, the algorithm clusters consecutive events together if the maximum distance from their centroid, computed as the median of the pings' latitudes and longitudes, is less than some roaming distance, d^{roam}. The first and last ping mark the start and end time of the stay. At least two subsequent events need to be observed within d^{roam} to be considered a stay. To better estimate the location of places that are visited frequently by the same user, the algorithm also checks whether different clusters appear within d^{roam} of each other and assigns a single consistent location to all connected clusters by recomputing their centroid. We use d^{roam} = 50m, and set the minimum duration of a stay to be 5 minutes.

2.4 Attributing stays to Points of Interest (POI)

A key feature of our analysis is how we model the attribution of stays detected in the mobility data to specific Points of Interest (POI), some of which are food outlets. Like stays, each POI is represented by a single point in space. We attribute each stay to the closest POI in our dataset (discussed below), calculated from the centroid of the user's stay to the centroid of the spatial polygon of the POI. This approach has been demonstrated to be accurate for inferring visited POI from passively collected smartphone mobility data[10]. To avoid attributing a

stay to a distant POI, we choose only the closest POI within a radius of a thresholding maximum distance, d^{max}, which we set at 200m. If a stay is further than d^{max} from any venue, the stay is discarded. Although we set d^{max}= 200m, the median distance of stays attributed to POI, d^{stay}, is 29.8 m (IQR, 14.7 - 55.4), and the median distance of a stay attributed to food outlet POIs (defined below) specifically is 27.2 m (IQR, 12.5 - 67.0).

2.5 Tests of robustness for attributing stays to POI

We tested the robustness of our results to this method for attributing stays to POI by comparing calculated mobility variables and study findings across values for d^{max} = 20, 50, 100, and 200m. Since the median accuracy of this data is approximately 21m, we do not explore values of d^{max} below 20m. **Supplementary Table 4** presents statistics on the total number of stays and the median (IQR) distance d^{stay} between a stay and attributed POI at each value of d^{max}. Applying a d^{max} of 20m or 50m is very restrictive, as evidenced by the low number of stays attributed and median values of d^{stay} under these conditions.

We perform robustness tests at two levels of analysis. First, we found the Pearson correlation (R) between the values of each FF outlet visit variable calculated at d^{max}=200 and all other values of d^{max}. The correlations between the two variables were very strong for all values of d^{max} and significant at P<.001. For FF visits/time, we find R[d^{max}=200m, d^{max}=100m] = 0.98, R[d^{max}=200m, d^{max}=50m] = 0.96, and R[d^{max}=200m, d^{max}=20m]=0.91; for FF visits/food we find R[d^{max}=200m, d^{max}=100m] = 0.99, R[d^{max}=200m, d^{max}=50m] = 0.97, and R[d^{max}=200m, d^{max}=20m] = 0.93. These results suggest that the FF outlet visit variables are largely independent of the details of our approach for attributing stays to POI.

We also tested the robustness of the results of the association between FF outlet visits and FF intake frequency to the choice of d^{max}. Specifically, we linked the FF outlet visit variables calculated at each d^{max} to the analytic sample of LACHS users based on estimated home LAC Neighborhood of residence, and re-fit regression models of the association between FF intake frequency and the FF visit variables for each value of d^{max}. Comparing results across values of d^{max}, the estimated effect of FF visits/time on FF intake frequency changed by less than 1.5%, and of FF visits/food of FF intake frequency by less than 3.5%, with all effects remaining highly significant (**Supplementary Table 5**). These results indicate that our main findings are largely independent of and robust to the details of our approach for attributing stays to POI.

3. Identifying Food and Fasts Food Outlets

We obtain the location of POI and food outlets in LAC using a large places database obtained from the technology company Foursquare via their Public Search API[11] in 2017 and according to their terms and conditions of use. Foursquare, now called Foursquare City Guide, popularized the concept of real-time location-sharing and checking-in[12]. The data is built from a combination of crowd-sourced user activity and the aggregation of data from additional sources[13]. A 2018 study comparing the Foursquare POI database with other public POI databases from mapping and social media platforms (Facebook, Foursquare, Google, Instagram, OSM, Twitter, and Yelp) established that while none of these databases is complete, Foursquare's data quality, as measured by number of POI, number of categories included, and positioning accuracy, was among the best[14].

The POI database we downloaded in 2017 provides the names and geolocation of n=239,509 POI in LAC. These POI came classified by Foursquare across ten primary categories, *pcat* (e.g., *pcat* = Food; Shop &

Service; Nightlife Spot; College & University; etc.) and 665 subcategories, *cat* (e.g., for *pcat*=Food, *cat* = Fast Food; American; Burger; Vietnamese; etc.)[1].

We took several approaches to modify and recode this existing taxonomy to define the categories of *food outlets* and *FF outlets* analyzed in this study. We define a *food outlet* as any location where food might be sold. We start by accepting all POI falling under Foursquare's existing primary category of *pcat=Food*. We then combed through all subcategories under a primary category not equal to *Food* (e.g., *pcat = Nightlife Spot*) to identify additional locations where food might be sold, which we re-coded as *food outlets*. **Supplementary Table 6** shows all the non-food primary category and subcategory combinations coded as *food outlets*.

To define the *FF outlet* category, we start by accepting all POI within the primary category *cat=Food* and subcategory *cat=Fast Food*. We enrich this Foursquare-defined list by performing a search of known chain FF outlets validated in previous nutritional health research as representing limited-service restaurants serving menus of predominantly ultra-processed and/or low-nutrient, energy dense foods[15,16], shown in **Supplementary Table 7**. The search was performed by matching substrings from the list in **Supplementary Table 6** with the names of POI in the Foursquare database. After re-coding, we find a total of 53,588 *food outlets* and 4,151 *FF outlets* in LAC out of the n=239,509 POI in the Foursquare database. In comparison, the LAC Restaurant and Market Inventory[17], which comprises Environmental Health permitted restaurants and markets in LAC that are inspected by the LAC Department of Public Health, contains a total of 40,600 restaurants and markets, approximately 13,000 fewer food outlets than the Foursquare database.

The Foursquare POI database we use has limitations, including coverage of food outlets that are infrequently visited and spatially dynamic outlets like food trucks. Yet, it is well-established that all food environment databases have limitations[18]. We have demonstrated that the Foursquare database is more comprehensive than the LACDPH-maintained inventory of permitted food-selling establishments[17]. Additionally, previous work has demonstrated that its quality was among the best of any publicly available POI database in 2018, close to the time of our data collection in 2017[14].

4. Los Angeles County Neighborhoods (LACN)

The LACN were designed as part of a community-sourced project to map LAC communities, led by the Los Angeles Times (LA Times) Newspaper Datadesk[19]. They were crafted with the goal of representing communities across LAC with more similar groups of people. Neighborhoods are classified into three "types": *segment-of-a-city*, *standalone-city*, and *unincorporated-area*. To define Neighborhoods across the county, the LA Times started with the U.S. Census Bureau's boundaries of 88 cities and 43 Census-designated places and worked with its readership to determine which cities to keep as a *standalone-city*, such as Santa Monica; and which to subdivide into multiple *segment-of-a-city*. The City of Los Angeles (population approximately 4 million, 40% of the LAC population) is divided into 114 *segment-of-a-city*. The *segment-of-a-city* units were created by joining neighboring Census tracts within the city boundaries through an iterative mapping process with the LA Times' readership. Census-designated places were either kept as independent areas and categorized as *unincorporated-area* units; or, if they closely adjoin or are entirely within a city boundary, were combined with those cities.

[1] For a list of all venue categories see https://developer.foursquare.com/docs/build-with-foursquare/categories/

The median (IQR, range) number of residents in the LACN is 27,499 (IQR, 12,961-53,124; range, 58-471,568). A map of the resulting LACN boundaries is provided in **Supplementary Figure 1**. The median (IQR) land area of the LACN in square miles (mi^2) is 3.63 (IQR, 1.84-8.78) mi^2, and the range is 0.26-442.00 mi^2.

5. Neighborhood-Level Aggregation of Mobility-Derived Variables

Mobility measures were aggregated and averaged across users within spatial areas. Aggregation to an area-level was necessary because privacy protections set out in the University of Southern California and the Massachusetts Institute of Technology IRB protocols did not allow reporting on the behavior of individual mobility users. We explored aggregating the users over two existing administrative spatial boundary divisions: the U.S. Census Bureau-defined Census tract level (n=2,622 within LAC); and the LAC Neighborhood (LACN) level (n=272). The Census tract level is a geographic region system designed by the U.S. Census Bureau to divide the population into relatively homogeneous units with respect to population characteristics and size.

We investigated the sufficiency of the mobility user sample size within boundaries at each level (subsection: Mobility User Sample Size Sufficiency), and whether broad population representativeness is achieved at each level (subsection: Population Representativeness). Based on the results discussed below, we decided to aggregate smartphone users at the LACN level. As a final step, we used post-stratification weighting to adjust the sampling of FF outlet visit variables within neighborhoods to appropriately represent the census tracts composing each LACN (subsection: Post-Stratification Sampling to Represent Neighborhood-Level Populations).

5.1 Mobility user sample size sufficiency

We investigated whether the sample size of mobility users within each boundary designation was large enough to achieve stable estimates of mobility measures. This was tested by comparing the distribution of the mobility user sample size across the Census tracts with the distribution of user sample sizes across the LACN (**Supplementary Figure 4**).

Of the 2,346 Census tracts in LAC, 2,266 had residing smartphone users in our sample (based on estimated home address) and did not have a rural designation[20]. The median (IQR, range) number of users per Census tract was 68 (IQR, 50-91; range, 12-323); 86% of census tracts had fewer than 100 users. Of the 272 LACN, 247 had residing smartphone users and were not composed of a majority of rural Census tracts. The median (IQR, range) number of users per neighborhood was 464 (IQR, 227- 902; range, 14-7,326); 9% of these LACN had fewer than 100 users.

We concluded that the small sample of smartphone users within the Census tracts jeopardizes the ability to reach a stable estimate of the FF outlet visit variables within these areas, especially when considering that only a subset of users within each area will have logged any observations of FF outlet visit behavior.

5.2 Population representativeness

We investigated whether broad geographic representation of the underlying population size at the Census tract vs. LACN level was achieved by the mobility user sample. This was tested by evaluating the Pearson correlation between the number of users in the mobility dataset and the underlying Census population as reported in the 2017 American Community Survey (ACS)[21]. LACN populations were calculated by summing

the population of all Census tracts falling within a LACN geographic boundary. **Supplementary Figure 5** shows scatter plots of the Census population vs. user population at the (**a**) Census tract and (**b**) LACN levels, respectively, along with the estimated correlation coefficient (R) and P-value for significance. We find that the Census population and number of mobility users are correlated at the Census tract level with R = 0.66 (P<.001), and at the LACN level with R = 0.97 (P<.001). These results suggest that the mobility data sample is highly representative of the overall population size across the LACN, and less so at the Census tract level.

Because of the small sample size of mobility users within the majority of Census tracts, and the fact that broad geographic representation of the mobility user sample was achieved over the LACN, we decided to aggregate smartphone users at the LACN level.

5.3 Post-stratification sampling to represent neighborhood-level populations

We furthermore address the representativeness of the data using post-stratification sampling[22]. Post-stratification is a sampling tool that uses weighting to adjust raw observational data to meet known population or demographic distributions. It is commonly used by Computational Social Science researchers in application to datasets with broad population coverage, including mobile phone data[2,23]. The essence of the technique is to divide the observed sample into post-strata, and then to compute a weight for each post-strata or case within a post-strata before bringing the post-strata back together to compute statistics for the overall sample.

We performed post-stratification sampling to appropriately represent the population of the Census tracts that compose each LACN. We estimate the arithmetic mean of the FF visit variables within each Census tract, and then find the overall mean for a LACN by weighting according to each tract's proportion of the population within the LACN.

Specifically, let CT_j be a census tract, $\overline{FF_i^{CT_j}}$ be the mean of a FF outlet visit variable calculated over CT_j, and NB_k be a LACN. Then we can find the post-stratified mean of the FF outlet visit variable within NB_k, $\overline{FF_i^{NB_k}}$, as

$$\overline{FF_i^{NB_k}} = \sum_{CT_j \in NB_k} \overline{FF_i^{CT_j}} \cdot \frac{\omega^{CT_j}}{\omega^{NB_k}},$$

where ω represents population size. **Supplementary Figure 6** shows how (**a**) FF visits/time and (**b**) FF visits/food calculated at the LACN level change when the post-stratified (i.e., weighted) values are used instead of the unweighted values.

Post-stratifying the sample may alleviate bias of the data if not appropriately representing the populations of the Census tracts composing the LACN. However, the correlation between the weighted and unweighted values is R $\simeq$ 0.99 for each FF outlet variable, suggesting that the unweighted data was already almost fully representative of the population sizes of each composing Census tract. Therefore, post-stratification sampling made very little impact on our analyses.

6. Mobility Data Representativeness

6.1 Representativeness of the mobility data at the level of individual POI

In previous published work on this mobility dataset, we investigated whether our method for detecting when a user spends time at a specific POI is accurate[2]. We devised a test of the ability of our data and attribution approaches to detect visits to an individual POI in a way that is representative of the overall population. The test compares the official attendance counts at games of the major professional sports leagues to the estimates of attendance using our data. Our own estimates were based on (i) the number of individuals that have a stay within the perimeter of the large stadium polygon perimeter between 3 hours before starting time to 3 hours after the game's completion, and (ii) the representativeness of the mobility data to the overall population size. This test was conducted across games of the National Football League (NFL), National Basketball Association (NBA), and National Hockey League (NHL) in LAC, as well as other cities in the U.S. We found that estimates of attendance computed using our data were extremely close to official attendance counts, indicating that this mobility data achieves good representation of visits to large POI.

6.2 Demographic representativeness

In previous published work on this mobility dataset, we investigated whether the user data is representative of the Census distribution of income[2]. We first estimated the income level for each smartphone user from their mobility behaviors, for example what types of POI they visited and how much time they spent there. We then compared the distribution of estimated income levels across our sample of smartphone users to the distribution of income at the Census block group (CBG) level (from the ACS). This test was conducted within LAC and other major U.S. cities. From this imputation procedure we estimated that our sample of users has an average income that is 8.6% higher than the ACS distribution. This suggests that our sample of mobility users has low bias towards income classes after estimating an income level for each user.

We have not repeated this test with other demographic variables such as race and ethnicity or gender for reasons of user privacy and protection; whether these types of demographic variables should be imputed from digital trace data is an active topic of debate and discussion in the field of Computational Social Science[24,25].

7. Constructing Mobility Variables

7.1 Temporal frequency of FF outlet visits (FF visits/time)

To maximize the amount of user activity that can be analyzed while accounting for gaps in observation over time, we define unique set of observation periods for each user, and define the FF outlet visit variables relative to this observation set. We separate all days into three time periods: morning (12:00am -10:59am), midday (11:00am - 3:59pm), and evening (4:00pm - 11:59pm), for a set of P possible daily periods; if there are d days in our observation set, we now have $|P| \leq 3d$. For each user i, we identify the subset of P in which i had at least one stay, denoting this P_i^{stay}. We then identify the subset of P_i^{stay} in which user i had at least one visit at a FF outlet, denoting this as P_i^{FF}. We find the temporal frequency of visits to FF outlets for user i, i.e. the FF visits/time variable specific to user i, FF_i^{time}, as $FF_i^{time} = \frac{P_i^{FF}}{P_i^{stay}}$. The theoretical range for FF_i^{time} is [0,1]; $FF_i^{time} = 1$ if user i is observed to visit a FF outlet during every possible daily period in which they have at least one stay.

We obtain the variable FF visits/time at the level of spatial area A, FF_A^{time}, as the mean of FF_i^{time} over all N users residing within A,

$$FF_A^{time} = \frac{1}{N}\sum_{i \in A} FF_i^{time}.$$

7.2 Relative frequency of FF outlet visits (FF visits/food)

To define the relative frequency of FF outlet visits variable, FF visits/food, we consider all observations of visits to food and FF outlets, and do not use the possible daily periods *P* designation. For user *i*, we define the total number of visits to food outlets as V_i^{food}. We define the total number of visits to FF outlets as V_i^{ff}. We find the relative frequency of FF outlet visits for user *i*, i.e., the FF visits/time variable specific to user *i*, FF_i^{food}, as $FF_i^{food} = \frac{V_i^{ff}}{V_i^{food}}$. The theoretical range for FF_i^{food} is [0,1], and is equal to 1 if all of user *i*'s food visits are to FF outlets.

We obtain the variable FF visits/food at the level of spatial area *A*, FF_A^{food}, as the mean of FF_i^{food} over all *N* users residing within *A*,

$$FF_A^{food} = \frac{1}{N}\sum_{i \in A} FF_i^{time}.$$

7.3 Average number of trips per day (trips/day)

Let T_i be the total number of trips that user *i* takes during the *d* days of observation. We find the average number of trips per day across all users *i* within area *A*, $\overline{T_A}$, as

$$\overline{T_A} = \frac{1}{N}\sum_{i \in A}\frac{T_i}{d}.$$

7.4 Scaling of mobility variables

All continuous variables $X(x)$ from the mobility data are rescaled between [0,10] to $X'(x')$ by min-max scaling using the formula,

$$X'(x') = 10\frac{x - \min(X)}{\max(X) - \min(X)}.$$

A one-unit increase in the scaled variable $X'(x')$represents a 10% increase in the unscaled observed variable $X(x)$.

8. Equations for Regression Models

$$\ln\frac{P(FF\ INTAKE\ FREQUENCY = j)}{P(FF\ INTAKE\ FREQUENCY = J)} = \beta_0 + \beta_1 X_j + \beta_2 AGE + \beta_3 GENDER + \beta_4 RACE + \beta_5 INCOME + \beta_6 EDUCATION$$

For categories $j \in INFREQUENT, MODERATE, FREQUENT$ and $J = NO\ INTAKE$ (reference).

$$\text{logit}(P(OBESE = 1)) = \beta_0 + \beta_1 X_j + \beta_2 AGE + \beta_3 GENDER + \beta_4 RACE + \beta_5 INCOME + \beta_6 EDUCATION$$
$$\text{logit}(P(DIABETES = 1)) = \beta_0 + \beta_1 X_j + \beta_2 AGE + \beta_3 GENDER + \beta_4 RACE + \beta_5 INCOME + \beta_6 EDUCATION$$

where $X_j \in \{FF\ VISITS/TIME, FF\ VISITS/FOOD, FF\ INTAKE\ FREQUENCY\}$.

Supplementary Table 1. Statistics of the Distribution of Individual Stays in the Mobility Data Across the Analytic Sample of Smartphone Users*

Quantile	Number of stays	Number of days with stays
**Min**	1	1
**25%**	93	34
**Median**	172	57
**75%**	320	90
**Max**	6,706	182
**Total**	63,299,255	16,009,417

* Across the analytic sample of n=243,644 users in the mobility data observed between October 1, 2016, and March 31, 2017, 182 days.

Supplementary Table 2. Logistic Regression Analyses of the Association Between Visits to Fast Food Outlets With Diet-Related Disease Adjusted for General Mobility Behavior[a]

		Obesity outcome		Diabetes outcome	
**Model**	**Variable[b]**	**AOR (95% CI)**	***P* value**	**AOR (95% CI)**	***P* value**
FF visits/time	FF visits/time	1.16 (1.12 – 1.21)	<.001	1.15 (1.09, 1.21)	<.001
FF visits/time and trips/day	FF visits/time	1.14 (1.10 – 1.19)	<.001	1.14 (1.08 – 1.21)	<.001
	Trips/day	1.11 (1.05 – 1.18)	<.001	1.07 (0.99 – 1.15)	.098
FF visits/food	FF visits/food	1.13 (1.10 – 1.17)	<.001	1.11 (1.07 – 1.16)	<.001
FF visits/food and trips/day	FF visits/food	1.12 (1.09 – 1.16)	<.001	1.11 (1.06 – 1.16)	<.001
	Trips/day	1.13 (1.07 – 1.19)	<.001	1.08 (1.00 – 1.17)	.041

Abbreviations: AOR, adjusted odds ratio; FF, fast food.

[a] Binary logistic regression models adjusted for demographics: age group, gender, race and ethnicity, educational level, and household income level.

[b] Each model estimated fast food intake frequency using the variable or combination of variables listed in this column as the primary independent variable.

Supplementary Table 3. Number of Census Tracts and LACHS Respondents Removed After Identifying Outliers in Census Tract Demographic Change Between 2011 and 2017[a]

Outlier detection method	N (%) census tracts removed	N (%) LACHS respondents removed
Method 1[b]	256 (11.0%)	676 (12.4%)
Method 2[c]	150 (6.7%)	652 (12.0%)

[a] Outliers were identified for variables representing the percentage of the population in the census tract: (i) living above 200% of the FPL, (ii) that is Hispanic or Latino, and (iii) that is Black or African American alone. For each outlier identification method, we removed the union of outlier census tracts across the three measures and the LACHS respondents they were linked to and re-computed the sample distribution characteristics.
[b] Outliers are identified by the Tukey method as values more than 1.5 times the interquartile range from each of the quartiles for a variable, i.e. upper outliers are values of the distribution > Q3 + 1.5*IQR and lower outliers are values < Q1 – 1.5*IQR.
[c] Outliers are identified as values of a variable above and below 2 standard deviations of the mean. For each method, we remove from the LACHS sample all respondents living in outlier census tracts. We then identified the union over outlier census tracts across the three measures, as some census tracts overlapped.

Supplementary Table 4. Statistics of Stays at Each Value of Thresholding Maximum Distance

	**Stays at any POI**				**Stays at food outlet POI**			
	**Threshold for maximum distance between smartphone user and Point of Interest, d^{max} (meters)**							
**Statistic**	**20**	**50**	**100**	**200***	**20**	**50**	**100**	**200***
Total number of stays	17,484,338	35,367,227	49,955,726	63,299,255	5,781,210	9,803,413	12,296,092	14,498,850
Distance from stay to POI, d^{stay} median (IQR)	6.3 (4.2, 8.2)	11.1 (6.9, 15.3)	20.2 (11.2, 32.2)	29.8 (14.7, 55.4)	10.3 (6.3, 14.7)	16.6 (9.1, 27.8)	21.6 (10.8, 43.0)	27.2 (12.5, 67.0)

Abbreviations: IQR, interquartile range.
* Results in the main paper were calculated at $d^{max} = 200m$.

Supplementary Table 5. Unadjusted Odds Ratios of Self-Reported Fast Food Intake in Robustness Analyses of the Attribution of Stays Detected in Mobility Data to Points of Interest[a]

	FF intake frequency, unadjusted OR (95% CI)											
	**Infrequent**				**Moderate**				**Frequent**			
	**Threshold for maximum distance between smartphone user and Point of Interest, d^{max} (meters)**											
**Model[b]**	**20**	**50**	**100**	**200[c]**	**20**	**50**	**100**	**200[c]**	**20**	**50**	**100**	**200[c]**
FF visits/time	1.12 (1.06, 1.18)	1.13 (1.06, 1.19)	1.13 (1.06, 1.19)	1.13 (1.06, 1.20)	1.25 (1.19, 1.31)	1.26 (1.19, 1.32)	1.25 (1.19, 1.32)	1.26 (1.19, 1.33)	1.33 (1.27, 1.39)	1.34 (1.28, 1.41)	1.33 (1.27, 1.40)	1.35 (1.28, 1.42)
FF visits/food	1.13 (1.07, 1.19)	1.12 (1.06, 1.17)	1.12 (1.06, 1.17)	1.12 (1.06, 1.17)	1.25 (1.19, 1.31)	1.23 (1.18, 1.29)	1.23 (1.17, 1.28)	1.22 (1.16, 1.27)	1.33 (1.27, 1.40)	1.30 (1.25, 1.36)	1.29 (1.24, 1.35)	1.28 (1.22, 1.33)

Abbreviations: OR, odds ratio; FF, fast food.

[a] Multinomial logistic regression models for fast food intake frequency across four frequency categories; reference group: never. Values of FF outlet visit variables calculated at various thresholds for the maximum distance between a smartphone user and Point of Interest, d^{max}, used in our model for attributing stays detected in the mobility trajectory data to a Point of Interest. We investigate values of d^{max} = *20m, 50m, 100m*, and *200m*. Values of the FF outlet visit variables, calculated at each d^{max} and aggregated over each neighborhood, were linked to the analytic sample of LACHS users based on home census tract of residence. Regression models were fit to the linked data at each threshold. *P* <.001 for all estimated odds ratios.

[b] Each model estimated fast food intake frequency using the fast food visit variable listed in this column as the primary independent variable.

[c] Results in the main paper were calculated at d^{max} = *200m*.

Supplementary Table 6. Non-Food Primary Category and Subcategory Combinations Coded as *food outlets* in the Foursquare dataset

**Primary Category, *pcat***	**Subcategory, *cat***
Nightlife Spot	American, Asian, Bar, BBQ, Beach Bar, Beer Bar, Beer Garden, Brewery, Burgers, Café, Cocktail, French, Hookah Bar, Hotel Bar, Italian, Japanese, Korean, Lounge, Mediterranean, New American, Pizza, Sake Bar, Seafood, Speakeasy, Sports Bar, Sushi, Tiki Bar, Whisky Bar, Wine Bar, Wine Shop, Winery, Convenience Store, Dive Bar, Gastropub, Liquor Store, Mexican, Pub, Restaurant, Steakhouse, Wings
College & University	Café, Coffee Shop
Arts & Entertainment	American, Café, Cocktail, Dive Bar, Piano Bar, Pub, Restaurant, Speakeasy, Bar, Lounge, Pizza
Outdoors & Recreation	Farmer's Market, American, Speakeasy
Professional & Other Places	American, Cafeteria, Coffee Shop, Wine Bar, Winery, Corporate Cafeteria, Corporate Coffee Shop
Shop & Service	American, Beer Store, Candy Store, Cheese Shop, Chocolate Shop, Convenience Store, Deli / Bodega, Desserts, Discount Store, Farmer's Market, Fish Market, Food & Drink, Fruit & VegaTable Store, Gourmet, Grocery Store, Health Food Store, Juice Bar, Mexican, Organic Grocery, Restaurant, Sandwiches, Smoothie Shop, Snacks, Street Food Gathering, Supermarket, Warehouse Store, Wine Bar, Wine Shop, Bakery, Butcher, Café, Herbs & Spices Store, Liquor Store
Travel & Transport	Food Truck, Hotel Bar

Supplementary Table 7. List of Fast Food Outlet Names

Deangelo Pizza	Angelo Sandwich Shop	Schlotzky	Hardee	Burger King
Papa Gino	Jack In The Box	Dairy Queen	KFC	El Pollo Loco
Dominos	Bob Big Boy	Sonic	Kentucky Fried Chicken	Carl's Jr. / Green Burrito
Godfather Pizza	In-N-Out Burger	Blimpie Subs And Salads	KFC/Taco Bell	Carl's Jr./Green Burrito
Papa John	Five Guys	Waffle House	Wienerschnitzel	Carl's Jr.
Pizza Hut	Bob's Big Boy	Chick-Fil-A	Fuddruckers	Green Burrito
Pizza Inn	Checkers Drive-In Restaurant	Whataburger	Long John Silver	Baja Fresh
Shakey Pizza	Bojangles	Del Taco	Arby	Waba Grill
Papa Murphy	Denny	In N Out Burger	Harvey	Wendy's
Little Caesars	Steak N Shake	Krystal	Subway	Arby's
Pizza Delight	Togo	Buffalo Wild Wings	Orange Julius	Bob's Big Boy
Pizza Pizza	Popeye	Cici Pizza	Taco Bell	Rally's
Chuck E Cheese Pizza	Church Chicken	Frisch Big Boy	Taco John	Yoshinoya
Mr Gatti Pizza	White Castle	Hungry Howie	Wendy	Weinerscnitzel
Round Table Pizza	Carl Jr	Rally Hamburgers	Mc Donald	Sonic Drive-In
TGI Friday's	Quizno	Rally's	Mcdonald's	

List of fast food outlet names validated in previous nutritional health research as representing limited-service restaurants serving menus of predominantly ultra-processed and/or low-nutrient, energy dense foods[15,16], and used to enrich Foursquare's existing food outlet categorization.

Supplementary Figure 1. Map of the spatial boundaries of the 272 Los Angeles County neighborhoods

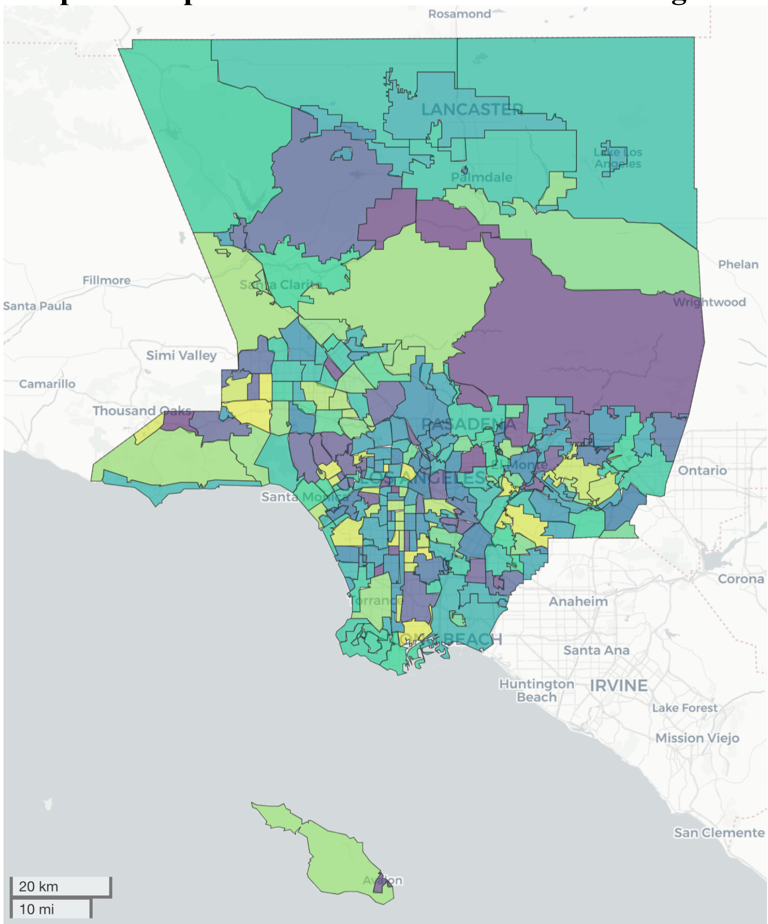

Map created using Leaflet for R with underlying map file coming from OpenStreetMap with contributors from CARTO, licensed under the Open Data Commons Open Database License (ODbL).

Supplementary Figure 2. Histograms of the Differences in Three Census Tract Variables Between 2011 and 2017

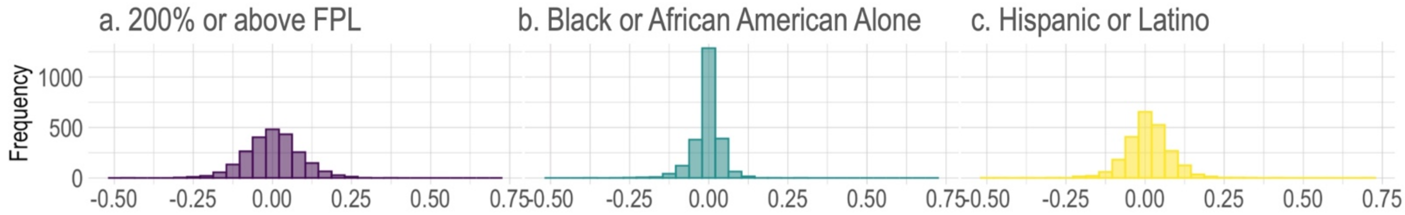

Histograms show the difference between 2011 and 2017 in the percent of the population in each LAC census tract above (a) 200% of the FPL, (b) that is Black or African American Alone, and (c) that is Hispanic or Latino.

Supplementary Figure 3. Frequency distribution of the accuracy of all pings generated by a random sample of 2,000 (0.8%) smartphone users in the mobility dataset

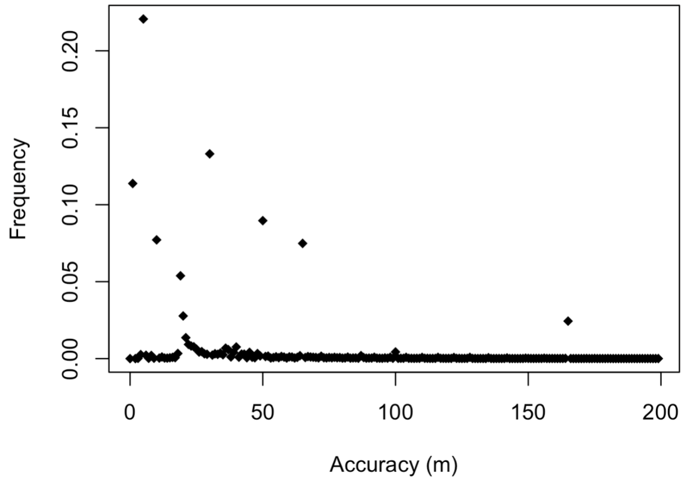

Abbreviation: meters, m.
Accuracy represents a specific level of ping GPS horizontal positioning accuracy in m. Frequency represents the share of the distribution that occurs at a specific level of accuracy. An accuracy of 0 means that the accuracy level of the ping is less than 1 m. Pings with accuracy > 200m were not included in analysis for this study.

Supplementary Figure 4. Distribution of the Number of Smartphone Users in Analytic Sample Within Each Area for the Census Tract and Neighborhood Spatial Levels

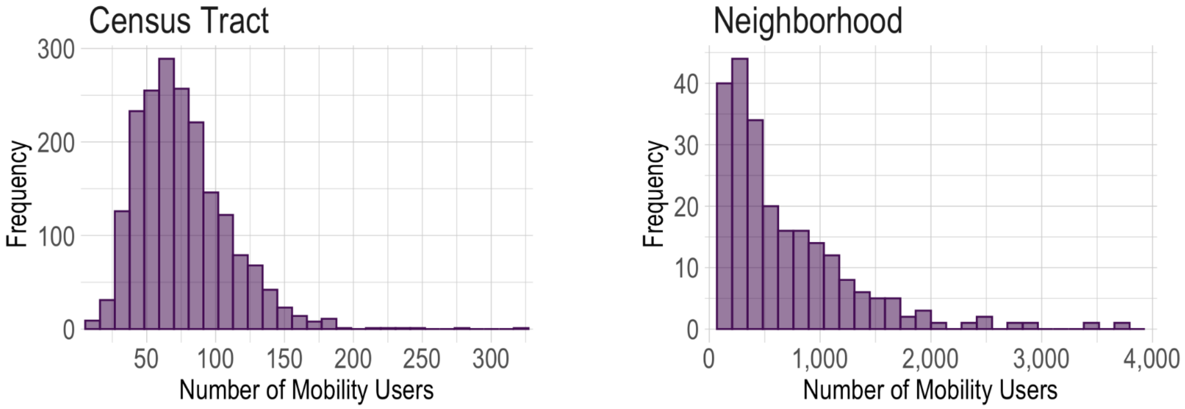

Supplementary Figure 5: Correlation Between the Size of the Smartphone User Population Detected in Mobility Data and the Size of the Census Population at the Census Tract vs. Los Angeles County Neighborhood Levels

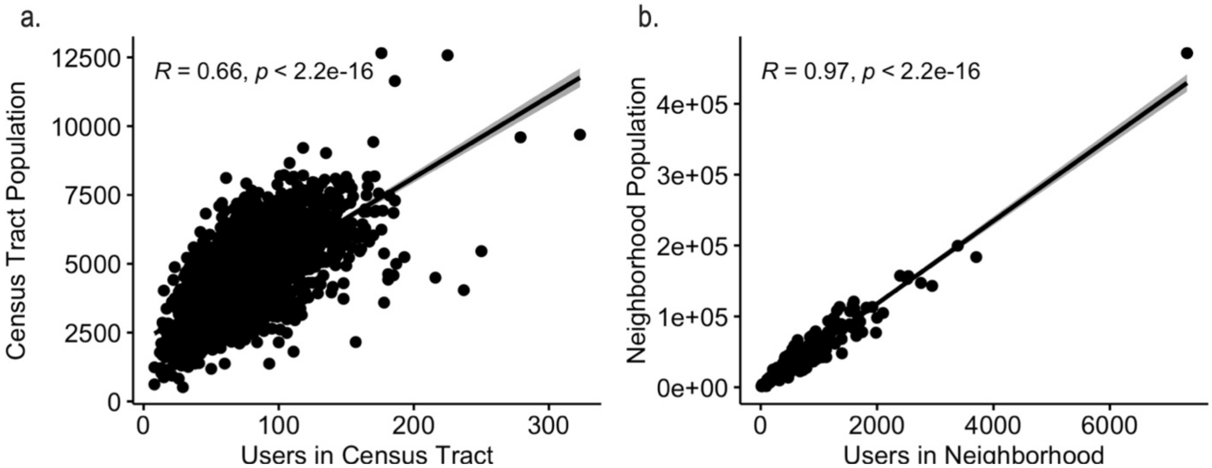

(a) Correlation of the size of the user population in each census tract detected in our smartphone data vs. size of the census population from the 2012-2016 ACS estimates. (b) Correlation of the size of the user population in each Los Angeles County Neighborhood detected in our smartphone data vs. size of the census population from the 2012-2016 ACS estimates.

Supplementary Figure 6. Correlation Between Post-Stratified (Weighted) and Unweighted Fast Food Outlet Visit Variables

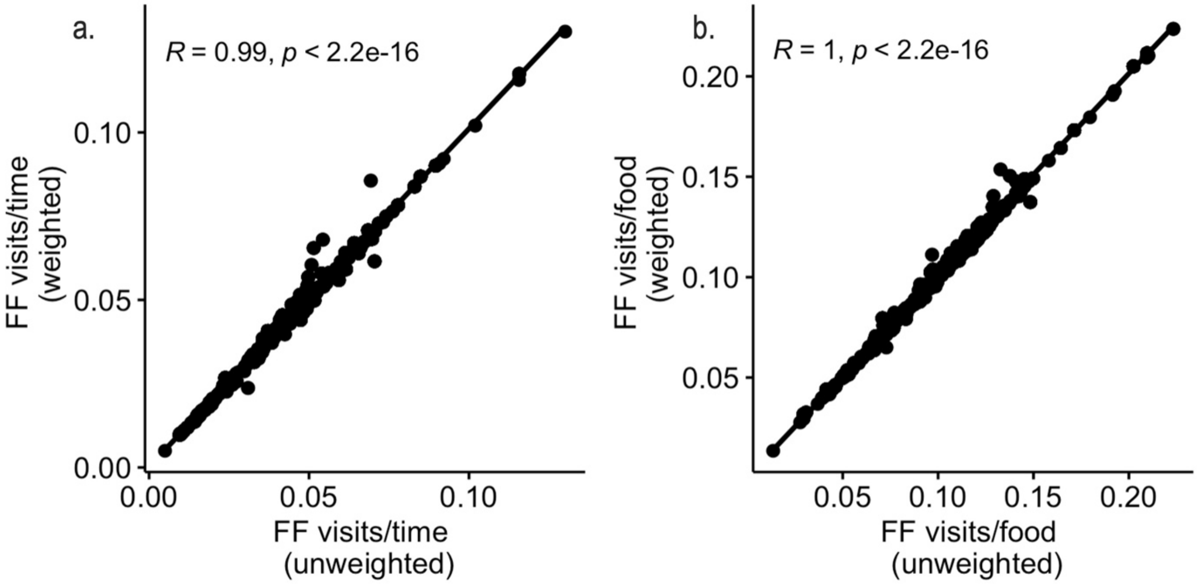

(a) Correlation of FF visits/time using the post-stratified (i.e., weighted) and unweighted values at the neighborhood level. (b) Correlation of FF visits/food using the weighted and unweighted values at the neighborhood level.

Supplementary References

1. National Institutes of Health. The practical guide: identification, evaluation, and treatment of overweight and obesity in adults. *US Dep. Heal. Hum. ...* (2000).
2. Moro, E., Calacci, D., Dong, X. & Pentland, A. Mobility patterns are associated with experienced income segregation in large US cities. *Nat. Commun.* **12**, (2021).
3. Aleta, A. *et al.* Modelling the impact of testing, contact tracing and household quarantine on second waves of COVID-19. *Nat. Hum. Behav.* **4**, (2020).
4. Spectus Social Impact Program. https://spectus.ai/social-impact.
5. Android Developer Reference: Location. https://developer.android.com/reference/android/location/Location.
6. Apple Developer: Horizontal Accuracy. https://developer.apple.com/documentation/corelocation/cllocation/1423599-horizontalaccuracy.
7. Merry, K. & Bettinger, P. Smartphone GPS accuracy study in an urban environment. *PLoS One* **14**, (2019).
8. Modsching, M., Kramer, R. & ten Hagen, K. Field trial on GPS Accuracy in a medium size city: the influence of built-up. *3rd Work. Positioning, Navig. Commun. 2006* **2006**, (2006).
9. Hariharan, R. & Toyama, K. Project Lachesis: Parsing and Modeling Location Histories. *Lect. Notes Comput. Sci. (including Subser. Lect. Notes Artif. Intell. Lect. Notes Bioinformatics)* **3234**, 106–124 (2004).
10. Cuttone, A., Larsen, J. E. & Lehmann, S. Inferring human mobility from sparse low accuracy mobile sensing data. in *UbiComp 2014 - Adjunct Proceedings of the 2014 ACM International Joint Conference on Pervasive and Ubiquitous Computing* (2014). doi:10.1145/2638728.2641283.
11. Foursquare API. https://developer.foursquare.com/.
12. Choudhury, S. R. Foursquare pioneered the trend of 'checking-in' to a place — now it sells access to its data to companies, CNBC. https://www.cnbc.com/2017/08/30/foursquare-pioneered-the-trend-of-checking-in-to-a-place--now-it-sells-your-data-to-companies.html.
13. Foursquare Places. https://foursquare.com/products/places.
14. Hochmair, H. H., Juhász, L. & Cvetojevic, S. Data quality of points of interest in selected mapping and social media platforms. in *Lecture Notes in Geoinformation and Cartography* vol. 0 (2018).
15. Datar, A. & Nicosia, N. Assessing social contagion in body mass index, overweight, and obesity using a natural experiment. *JAMA Pediatr.* **172**, (2018).
16. Datar, A., Mahler, A. & Nicosia, N. Association of Exposure to Communities With High Obesity With Body Type Norms and Obesity Risk Among Teenagers. *JAMA Netw. open* **3**, (2020).
17. Los Angeles County Department of Public Health. County of Los Angeles Restaurant and Market Inventory. https://data.lacounty.gov/Health/COUNTY-OF-LOS-ANGELES-RESTAURANT-AND-MARKET-INVENT/jf4j-8it9.
18. Fleischhacker, S. E., Evenson, K. R., Sharkey, J., Pitts, S. B. J. & Rodriguez, D. A. Validity of secondary retail food outlet data: A systematic review. *Am. J. Prev. Med.* **45**, (2013).
19. The Los Angeles Times Datadesk. Mapping L.A. Neighborhoods. http://maps.latimes.com/neighborhoods/.
20. United States Department of Agriculture Economic Research Service. Rural-Urban Commuting Area Codes (Updated 7/3/2019). https://www.ers.usda.gov/data-products/rural-urban-commuting-area-codes.aspx (2014).
21. United States Census Bureau. 2017 American Community Survey 5-Year Data. https://www.census.gov/ programs-surveys/acs.
22. Salganik, M. *Bit by bit: Social research in the digital age*. (Princeton University Press, 2019).
23. Jiang, S. *et al.* The TimeGeo modeling framework for urban motility without travel surveys. *Proceedings of the National Academy of Sciences of the United States of America* vol. 113 (2016).
24. Stern, A. *et al. Ethics and Empathy in Using Imputation to Disaggregate Data for Racial Equity, A Case Study Imputing Credit Bureau Data*. https://www.urban.org/research/publication/ethics-and-empathy-using-imputation-disaggregate-data-racial-equity-case-study-imputing-credit-bureau-data/view/full_report (2021).
25. Lazer, D. *et al.* Meaningful measures of human society in the twenty-first century. *Nature* vol. 595 (2021).
