# Supplementary material for: Population mobility data provides meaningful indicators of fast food intake and diet-related diseases in diverse populations
Source: NPJ Digit Med. 2023 Nov 15;6:208. doi: 10.1038/s41746-023-00949-x (PMC10651929; doi:10.1038/s41746-023-00949-x)
Supplement: Supplementary file 2 — Reporting Summary [file 41746_2023_949_MOESM2_ESM.pdf]

nature portfolio

Corresponding author(s): Abigail Horn

Last updated by author(s): 9/24/2023

Reporting Summary

Nature Portfolio wishes to improve the reproducibility of the work that we publish. This form provides structure for consistency and transparency in reporting. For further information on Nature Portfolio policies, see our Editorial Policies and the Editorial Policy Checklist.

Statistics

For all statistical analyses, confirm that the following items are present in the figure legend, table legend, main text, or Methods section.

n/a	Confirmed	
☐	☒	The exact sample size (*n*) for each experimental group/condition, given as a discrete number and unit of measurement
☒	☐	A statement on whether measurements were taken from distinct samples or whether the same sample was measured repeatedly
☐	☒	The statistical test(s) used AND whether they are one- or two-sided<br>*Only common tests should be described solely by name; describe more complex techniques in the Methods section.*
☐	☒	A description of all covariates tested
☐	☒	A description of any assumptions or corrections, such as tests of normality and adjustment for multiple comparisons
☐	☒	A full description of the statistical parameters including central tendency (e.g. means) or other basic estimates (e.g. regression coefficient) AND variation (e.g. standard deviation) or associated estimates of uncertainty (e.g. confidence intervals)
☒	☐	For null hypothesis testing, the test statistic (e.g. *F*, *t*, *r*) with confidence intervals, effect sizes, degrees of freedom and *P* value noted<br>*Give P values as exact values whenever suitable.*
☒	☐	For Bayesian analysis, information on the choice of priors and Markov chain Monte Carlo settings
☒	☐	For hierarchical and complex designs, identification of the appropriate level for tests and full reporting of outcomes
☐	☒	Estimates of effect sizes (e.g. Cohen's *d*, Pearson's *r*), indicating how they were calculated

Our web collection on statistics for biologists contains articles on many of the points above.

Software and code

Policy information about availability of computer code

Data collection	The LACHS health survey data were organized using SAS and Excel.
Data analysis	Mobility data were analyzed in Python; LACHS data and statistical analyses were conducted using R software, version 3.6.3.

For manuscripts utilizing custom algorithms or software that are central to the research but not yet described in published literature, software must be made available to editors and reviewers. We strongly encourage code deposition in a community repository (e.g. GitHub). See the Nature Portfolio guidelines for submitting code & software for further information.

Data

Policy information about availability of data

All manuscripts must include a data availability statement. This statement should provide the following information, where applicable:

- Accession codes, unique identifiers, or web links for publicly available datasets
- A description of any restrictions on data availability
- For clinical datasets or third party data, please ensure that the statement adheres to our policy

Mobility data are available from Spectus, available upon request submitted to https://spectus.ai/social-impact. The LACHS health survey data are available to investigators upon request and pending eligibility to access data governed by the Los Angeles County Department of Public Health Institutional Review Board. Other data used come from the 2017 5-Year American Community Survey, available at https://www.census.gov/programs-surveys/acs.

Research involving human participants, their data, or biological material

Policy information about studies with human participants or human data. See also policy information about sex, gender (identity/presentation), and sexual orientation and race, ethnicity and racism.

Reporting on sex and gender	We use secondary source data collected in the 2011 Los Angeles County Health Survey (LACHS). Gender was recorded by LACHS as self-reported male or female, used as the "gender" variable in this study (more recent versions of the LACHS survey include more categories for gender). The LACHS includes a variable for "gender" but not "sex". This was the main reason why gender was used in this study. Additionally, this study focuses on eating behaviors, which are shaped by social and cultural circumstances and thus may have stronger relationships with individuals' "gender" than to their "sex". The study included 4,863 (60.5%) individuals of self-identified female gender and 3,173 (39.5%) of self-identified male gender.
Reporting on race, ethnicity, or other socially relevant groupings	A joint race/ethnicity variable was created by the Los Angeles County Health Survey (LACHS) and used in data analysis in this study. The LACHS created the variable from survey questions that participants were instructed to self-report. We changed the name of the "race/ethnicity" variable provided by the LACHS survey to "race and ethnicity," as this is recommended in the guidance provided in the viewpoint, Updated Guidance on the Reporting of Race and Ethnicity in Medical and Science Journals (Flanagin et al., JAMA Network Open, 2021). The ethnicity component of the variable collected by the LACHS focused on Hispanic origin. Specifically: Race was measured by asking participants, "What is your race?" (White; Black/African American; Asian; Pacific Islander; American Indian/Alaskan Native; Hispanic/Latino; Other; Do not know; Refused). Hispanic origin was measured by asking participants, "Are you of Latino or Hispanic origin?" (yes, no). These two variables were coded by LACHS into a combination variable with 7 categories: Hispanic/Latino; White; Asian; Black/African American; Native Hawaiian or Pacific Islander; American Indian/Alaskan Native; Other, based on the following rules:<br>* If Hispanic/Latino mentioned at all, Hispanic/Latino was assigned.<br>* Else if Black/African American mentioned at all, African American was assigned.<br>* Else if Pacific Islander mentioned at all, Native Hawaiian or Other Pacific Islander (NHOPI) was assigned.<br>* Else if Asian mentioned at all, Asian was assigned.<br>* Else if White mentioned only, White was assigned.<br>* Else if American Indian/Alaska Native mentioned only, American Indian/Alaska Native (AI/AN) was assigned<br>* The remaining was assigned as Other.<br>We re-coded this 7-combination variable into the 5-category "race and ethnicity" variable used in this study: Hispanic/Latino; White; African American; Asian; and Multiracial/Other, which includes Pacific Islander, American Indian/Alaskan Native, do not know, and refused. The latter four categories were recoded into a single "Multiracial/Other" category because they individually made up a small percentage of the overall sample (2.2%), facilitating statistical analysis.
Population characteristics	See below.
Recruitment	The LACHS is a population-based dual frame (landline and cellular) telephone survey conducted by the Los Angeles County (LAC) Department of Public Health (LACDPH), using a stratified sampling approach to collect data from samples of adults living within LAC designed to be representative of the overall LAC population demographics, as well as the demographics in 8 Service Planning Areas (SPAs) within LAC. Detailed study protocols are available from LACHS: http://www.publichealth.lacounty.gov/ha/hasurveyintro.htm. Self-selection bias is limited due to the randomization of the telephone survey approach.<br>The mobility data were collected by Spectus by aggregating data across smartphone applications from mobile phone devices. The dataset consists of anonymized records of GPS locations from a convenience sample of individual adult (≥18) smartphone users who have opted in to provide access to their GPS location data anonymously through a General Data Protection Regulation and California Consumer Privacy Act compliant framework. Users across all major smartphone device operating systems (e.g., iOS, Android, Windows) are represented. There may be bias in the smartphone user population. Although smartphone users constituted 83% of the U.S. adult population in 2017, they represent a subset of the population that has some uneven representation across socio-demographic groups (e.g., low income, older and non-white). Quantifying these biases in mobility data is challenging since demographic information is not available on individual smartphone users to protect privacy. We have taken several steps to investigate and address the representativeness of the sample to the overall population: (i) we establish that our sample sizes are representative of the population size in each LACN, and (ii) in our previous work on this dataset, we demonstrated low bias across income classes by imputing this characteristic for each user based on their mobility-observed shopping behaviors.
Ethics oversight	All study protocols were approved by the Institutional Review Boards (IRB) of the Los Angeles County Department of Publich Health, the University of Southern California, and the Massachusetts Institute of Technology

Note that full information on the approval of the study protocol must also be provided in the manuscript.

Field-specific reporting

Please select the one below that is the best fit for your research. If you are not sure, read the appropriate sections before making your selection.

☐ Life sciences ☒ Behavioural & social sciences ☐ Ecological, evolutionary & environmental sciences

For a reference copy of the document with all sections, see nature.com/documents/nr-reporting-summary-flat.pdf

Behavioural & social sciences study design

All studies must disclose on these points even when the disclosure is negative.

Study description	This study is a secondary analysis of data on adult residents of Los Angeles County (LAC), California, USA. A representative sample of LAC residents who participated in the 2011 Los Angeles Community Health Survey (LACHS) from the LAC Department of Public Health was used to obtain cross-sectional measures of individual-level self-reported frequent fast-food intake (>1/week); obesity; diabetes type 2 diagnosis; and respondents' sociodemographics (age group, gender, race/ethnicity, education level, and household income level) and census tract of residence. A large-scale, individual-level mobility dataset representing all geolocations between October 2016 - March 2017 of a convenience sample of anonymous adult smartphone users residing in LAC was obtained from Spectus, a location aggregation company. Variables representing visits to food outlets aggregated over mobility users within a neighborhood were linked as contextual variables to individual respondents from the LACHS. The objective was to investigate whether FF outlet visits from mobility data are indicators of self-reported FF intake, obesity, and diabetes, and compared with self-reported intake, equivalent or better indicators of obesity and diabetes.
Research sample	The research sample involved two secondary data sources: (1) a representative sample of 8,036 adult residents of Los Angeles County (LAC) from the 2011 Los Angeles County Health Survey (LACHS), and (2) mobility data representing all geolocations between October 2016 - March 2017 of a convenience sample of 243,644 anonymous and opted-in smartphone users in LAC, collected by the mobility data company Spectus, a location-based services company that maintains anonymized geospatial datasets on human mobility by aggregating data across smartphone applications from mobile phone devices. The analytic sample included 5,447 LACHS respondents and 234,995 smartphone users with 14,498,850 visits to food outlets. Variables representing visits to food outlets aggregated over mobility users within a neighborhood were linked as contextual variables to individual respondents from the LACHS. The LACHS sample was chosen because it provided individual variables on fast food intake and diet-related disease, necessary for analysis of the objective of this study. The mobility data from Spectus were chosen because of their high-resolution and ability to capture refined measures of visits to food outlets over individual users. The mobility variables were aggregated into measures at an area level because privacy protections set out in the IRB protocols did not allow reporting of individual mobility user behavior. Aggregation was therefore the only way to relate measures of visits to food outlets to individual respondents in the LACHS health survey sample.
Sampling strategy	The LACHS is a population-based dual frame (landline and cellular) telephone survey conducted by the Los Angeles County (LAC) Department of Public Health (LACDPH), using a stratified sampling approach to collect data from samples of adults living within LAC designed to be representative of the overall LAC population demographics, as well as the demographics in 8 Service Planning Areas (SPAs) within LAC. Detailed study protocols are available from LACHS: http://www.publichealth.lacounty.gov/ha/hasurveyintro.htm<br>The mobility data were collected by Spectus by aggregating data across smartphone applications from mobile phone devices. The dataset consists of anonymized records of GPS locations from a convenience sample of individual adult (≥18) smartphone users who have opted in to provide access to their GPS location data anonymously through a General Data Protection Regulation and California Consumer Privacy Act compliant framework. Users across all major smartphone device operating systems (e.g., iOS, Android, Windows) are represented.
Data collection	LACHS survey data were collected by survey coders who input answers from survey respondents shared over phone into an electronic form.<br>The mobility data were collected via software developed by Spectus capable of aggregating data from individual users across multiple mobile applications.
Timing	The LACHS survey data were collected throughout 2011. The mobility data were collected between October 2016 - March 2017.
Data exclusions	We excluded LACHS participants who: were missing residential census tract information (meaning we could not link mobility variables to these respondents), lived in a rural census tract (meaning we were unable to define mobility variables in these areas), or had missing data for all outcome variables (meaning we could not include them in statistical models). The original sample of LACHS respondents consisted of 8,036 individuals, while the final analytic sample was 5,447 participants after the above exclusions.<br><br>We excluded mobility users if they had fewer than two stays at any location over the 6 months, resulting in a final analytic sample of 234,995 users, down from 243,644 in the original sample.
Non-participation	N/A
Randomization	N/A

Reporting for specific materials, systems and methods

We require information from authors about some types of materials, experimental systems and methods used in many studies. Here, indicate whether each material, system or method listed is relevant to your study. If you are not sure if a list item applies to your research, read the appropriate section before selecting a response.

Materials & experimental systems

n/a	Involved in the study	
☒	☐	Antibodies
☒	☐	Eukaryotic cell lines
☒	☐	Palaeontology and archaeology
☒	☐	Animals and other organisms
☒	☐	Clinical data
☒	☐	Dual use research of concern
☒	☐	Plants

Methods

n/a	Involved in the study	
☒	☐	ChIP-seq
☒	☐	Flow cytometry
☒	☐	MRI-based neuroimaging
